# The m^6^A pathway facilitates sex determination in *Drosophila*

**DOI:** 10.1038/ncomms15737

**Published:** 2017-07-04

**Authors:** Lijuan Kan, Anya V. Grozhik, Jeffrey Vedanayagam, Deepak P. Patil, Nan Pang, Kok-Seong Lim, Yi-Chun Huang, Brian Joseph, Ching-Jung Lin, Vladimir Despic, Jian Guo, Dong Yan, Shu Kondo, Wu-Min Deng, Peter C. Dedon, Samie R. Jaffrey, Eric C. Lai

**Affiliations:** 1 Department of Developmental Biology, Sloan-Kettering Institute, 1275 York Ave, Box 252, New York City, New York 10065, USA; 2 Department of Pharmacology, Weill Medical College, Cornell University, New York City, New York 10065, USA; 3 Department of Biological Engineering, Massachusetts Institute of Technology, Cambridge, Massachusetts 02139, USA; 4 Department of Biological Science, Florida State University, Tallahassee, Florida 32306, USA; 5 Key Laboratory of Insect Developmental and Evolutionary Biology, Institute of Plant Physiology and Ecology, Shanghai Institutes for Biological Sciences, Chinese Academy of Sciences, Shanghai 200032, China; 6 University of Chinese Academy of Sciences, Beijing 100049, China; 7 Invertebrate Genetics Laboratory, National Institute of Genetics, Mishima, Shizuoka 411-8540, Japan

## Abstract

The conserved modification *N*^6^-methyladenosine (m^6^A) modulates mRNA processing and activity. Here, we establish the *Drosophila* system to study the m^6^A pathway. We first apply miCLIP to map m^6^A across embryogenesis, characterize its m^6^A ‘writer’ complex, validate its YTH ‘readers’ CG6422 and YT521-B, and generate mutants in five m^6^A factors. While m^6^A factors with additional roles in splicing are lethal, m^6^A-specific mutants are viable but present certain developmental and behavioural defects. Notably, m^6^A facilitates the master female determinant *Sxl*, since multiple m^6^A components enhance female lethality in *Sxl* sensitized backgrounds. The m^6^A pathway regulates *Sxl* processing directly, since miCLIP data reveal *Sxl* as a major intronic m^6^A target, and female-specific *Sxl* splicing is compromised in multiple m^6^A pathway mutants. YT521-B is a dominant m^6^A effector for *Sxl* regulation, and YT521-B overexpression can induce female-specific *Sxl* splicing. Overall, our transcriptomic and genetic toolkit reveals *in vivo* biologic function for the *Drosophila* m^6^A pathway.

## Abstract (web summary)

*N* ^6^-methyladenosine (m^6^A) is a conserved RNA modification that has recently emerged as an important regulator of messenger RNA processing and activity. Here, the authors provide evidence that m6A pathway facilitates female-specific splicing of *Sxl*, regulating sex determination in *Drosophila*.

Of the ∼100 post-transcriptional chemical modifications to RNA known, *N*^6^-methyladenosine (m^6^A) is the most prevalent modification in messenger RNA (mRNA)^1^. Although methylated mRNA was described over 40 years ago^2^,^3^, understanding the biological roles of m^6^A during most of this time was limited by lack of knowledge of molecular mechanisms that generate and interpret this modification, uncertainty on identities of methylated transcripts, and paucity of genetic mutants in specific m^6^A pathway components that could reveal *in vivo* requirements of this nucleoside modification^4^. However, the past few years have witnessed remarkable progress on all of these fronts^5^.

While METTL3 was identified as the central catalytic subunit of the large mammalian m^6^A-methyltransferase complex (MTC) some time ago^6^, other components in the ‘writer’ complex were appreciated more recently. The annotated methyltransferase factor METTL14 and the splicing regulators WTAP, KIAA1429 and RBM15/RBM15B also associate with the MTC and are functionally required for normal accumulation of m^6^A (refs 7, 8, 9, 10, 11). Recent structural studies clarify that METTL14 binds directly to METTL3, but lacks methyltransferase activity and instead serves a structural role in the MTC^12^,^13^,^14^. WTAP may scaffold the MTC and RBM15/RBM15B recruits the MTC to target sites of modification. Reciprocally, the identification of m^6^A demethylase ALKBH5^15^,^16^ indicates that methylation can be ‘erased’. m^6^A marks are bound by different ‘readers’, notably including by factors bearing YTH domains (for example, YTHDC1/2 and YTHDF1/2/3)^9^,^17^,^18^,^19^.

The m^6^A writers, erasers and readers comprise a system for epitranscriptomic regulation, akin to chromatin-based epigenetic systems. This framework is bolstered by transcriptomewide mappings of methylated regions^19^,^20^,^21^, catalogues that were refined by identification of specifically methylated adenosines^22^,^23^,^24^. Diverse functional consequences of m^6^A and/or m^6^A factors have been reported, including by promoting mRNA splicing^8^,^19^, pri-miRNA processing^25^, affecting RNA structure^26^, facilitating mRNA degradation^10^, promoting expression^27^ and/or canonical translation^28^, and promotion of cap-independent translation^29^,^30^.

Most studies of m^6^A biology and mechanism have been performed in yeast and in mammalian cell culture. The *Drosophila* system offers attractive possibilities for combining molecular, genetic and genomic approaches to studying mRNA methylation, especially within tissue-specific settings that are not well-addressed by cell models. However, few studies of the m^6^A pathway have yet been performed in flies. One study reported the *Drosophila* METTL3 ortholog dIME4 is essential for viability, and that hypomorphic mutants displayed aberrant ovarian organization and sterility^31^. Beyond this, the *Drosophila* orthologs of mammalian m^6^A ‘writer’ factors WTAP, KIAA1429 and RBM15/RBM15B were previously identified in *Drosophila* as Fl(2)d^32^, Virilizer^33^ and Spenito (Nito)^34^, respectively. Interestingly, these genes affect a common regulatory pathway, as all three are required for female-specific splicing of the master sex determination factor Sex-lethal (Sxl)^35^,^36^. Fl(2)d and Virilizer form a complex to regulate alternative splicing^37^, similar to their mammalian homologues^9^. These findings suggest the possibility that sex determination might be linked to m^6^A modifications.

Here, we generate a genomic and genetic toolkit to explore the m^6^A pathway in *Drosophila*. In particular, we produce single-nucleotide resolution maps of methylated transcripts, characterize the *Drosophila* methyltransferase complex, validate *Drosophila* YTH factors as m^6^A readers, and use CRISPR/Cas9 to mutagenize the writer and reader machinery. Mutants in dedicated m^6^A pathway components are viable but exhibit specific gonadal and behavioural phenotypes. Notably, we demonstrate that m^6^A writer factors and YTH readers, particularly YT521-B, facilitate female-specific splicing of *Sxl*, highlighting sex determination as a substantial *in vivo* process that involves RNA methylation.

## Results

### Single nt resolution mapping of m^6^A across fly embryogenesis

Methyl-RNA immunoprecipitation and sequencing using m^6^A-specific antibodies has been utilized to map m^6^A-modified transcripts in different species^4^. Recently, we showed that m^6^A individual-nucleotide-resolution cross-linking and immunoprecipitation (miCLIP) induces specific mutational signatures that precisely identify methylated adenosines^23^. We applied miCLIP to six embryo developmental stages that interrogate the maternal transcriptome (T1: 0–45′ and T2: 45–90′), and successive timepoints following activation of zygotic transcription (T3: 1.5–6 h, T4: 6–12 h, T5: 12–18 h and T6: 18–24 h). The miCLIP libraries were sequenced to depths of ∼14–50 million mapped reads (Supplementary Data 1) and subjected to our pipeline for identification of cross-linking–induced mutation sites (CIMSs).

**Figure 1:**
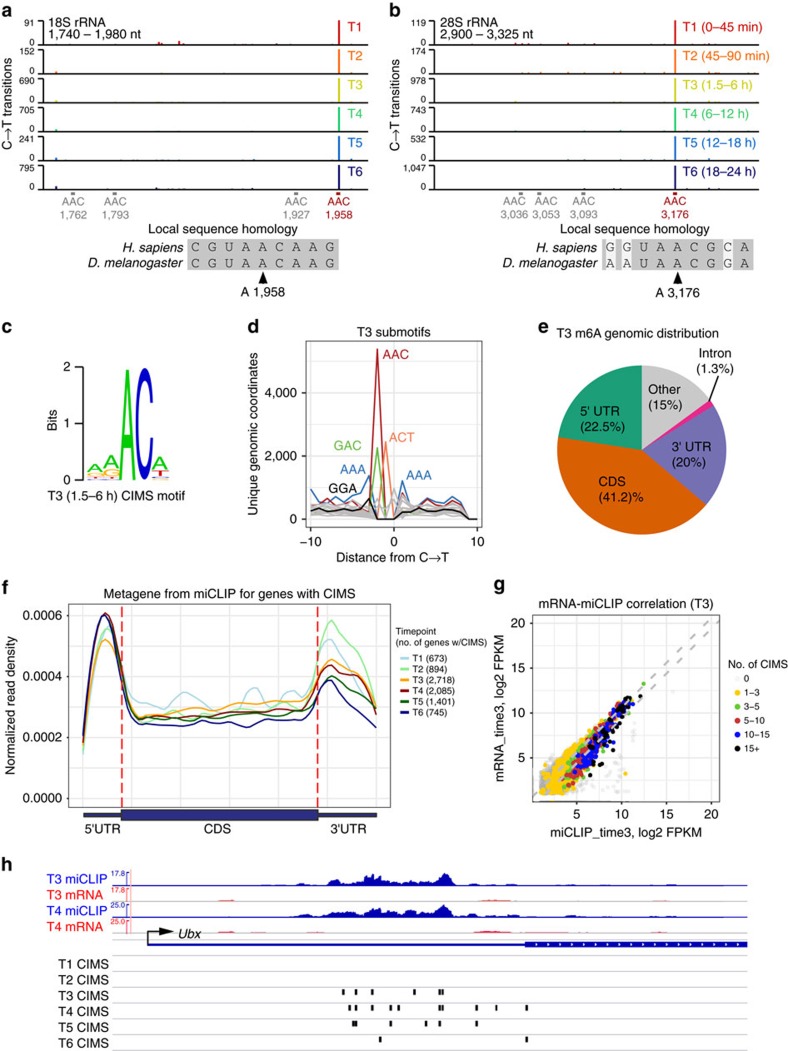
miCLIP-seq analysis of *Drosophila* embryogenesis. (**a**,**b**) miCLIP analysis at 18S rRNA (**a**) and 28S rRNA (**b**). Y-axis shows number of reads with C→T cross-linking–induced mutation site (CIMS) calls in 6 developmental miCLIP libraries across fly embryogenesis (T1-T6). The positions of A1958 in 18S rRNA and A3176 in 28S are indicated as highly modified m^6^A sites that are conserved between fly and human. For reference, other ‘AAC’ contexts that were not recovered in miCLIP-seq are indicated, demonstrating specificity of this modification. (**c**) Nucleotide bias surrounding CIMS calls preceded by A. Shown is the motif obtained using timepoint 3 (1.5–6 h embryos). (**d**) Positional enrichment of submotifs in the vicinity of CIMS calls. The C→T transitions are centred at 0, and the frequency of triplet motifs at all positions is plotted. Select motifs are positionally enriched near CIMS calls. For example, there is a two-fold enrichment of ‘GGA’ in the three nucleotides upstream of C→T transitions. These support that a DRACH-like consensus is enriched at CIMS, with an overall A bias. Analysis of other timepoints is shown in Supplementary Fig. 2. (**e**) Genomic annotations of CIMS/m^6^A sites in timepoint three. Analysis of other timepoints is shown in Supplementary Fig. 3. (**f**) Metagene plot of miCLIP data at genes with CIMS in the six libraries that span *Drosophila* embryogenesis. There is enrichment in untranslated regions, including in the vicinity of stop codons. (**g**) Correlation of miCLIP and mRNA-seq data for timepoint 3; analysis of other timepoints is shown in Supplementary Fig. 4. (**h**) Example of *Ubx* as a locus with preferential miCLIP over RNA-seq, and bearing a cluster of reproducible CIMS calls within its 5′ UTR. *Ubx* is zygotically expressed and only two representative timepoints are shown for expression/miCLIP tracks; CIMS calls are shown for all 6 timepoints.

C→T transitions are enriched at genuine m^6^A sites in human miCLIP data^23^. Similarly, amongst various nucleotide substitution types in fly miCLIP data, only C→T transitions were preferentially enriched in preceding A residues (Supplementary Fig. 1). We subsequently filtered these calls for known *D. melanogaster* SNPs (Supplementary Data 2). To illustrate the utility of miCLIP, we analysed ribosomal RNAs. As we recently showed that miCLIP specifically recovers the known m^6^A sites in human 18S and 28S rRNA^23^, we surveyed the homologous regions of *Drosophila* rRNAs. Our miCLIP data recovered signatures for highly modified adenosines in AA*C triplets (where A* denotes methylated adenosine) in both *Drosophila* 18S and 28S rRNA (Fig. 1a,b). The sequence contexts were conserved between fly and human rRNA subunits, and these modifications were specific since other AAC sites in the vicinity lacked CIMS (Fig. 1a,b). Altogether, these data provide evidence that miCLIP of *D. melanogaster* RNA results in C→T transitions that can identify specific m^6^A sites.

In other species, m^6^A is enriched in local consensus motifs such as RAC/DRACH^4^. A majority of fly CIMS sites are preceded by A (*P*<0.0002), and of these ‘AC’ sites, a majority fell into an RAC context (62–68% across libraries). Accordingly, we observe statistical enrichment for RAC (*P*<0.00004) and DRACH (*P*<0.0001) at CIMS sites across all libraries (Supplementary Data 2). We note the nucleotides surrounding CIMS are not as strongly biased as in mammalian RNA subjected to miCLIP^23^. De novo motif analysis at CIMS preceded by A yielded mild enrichment of a DRACH-like consensus (Fig. 1c). The motifs differed from those in mammals in that m^6^A site contexts showed more A than G in the ‘DR’ positions. This may suggest a slightly different context for m^6^A in *Drosophila*. Nevertheless, we still observe positional enrichment of GGA and GAC at CIMS sites (Fig. 1d and Supplementary Fig. 2), indicating a bias towards the core context seen in other species. Overall, the CIMS preceded by adenosine, filtered for SNPs, define a catalogue of 21892 methylated sites that includes 4501 genes (Supplementary Data 3). Details of CIMS calls that map to annotated genes and their locations are provided in Supplementary Data 4.

The bulk of CIMS calls in different timepoints were located in coding sequences (Fig. 1e and Supplementary Fig. 3). However, as UTRs occupy less space in the transcriptome, metagene plots of miCLIP reads at CIMS sites indicate relative m^6^A enrichment in 5′ UTRs and 3′ UTRs relative to coding regions (Fig. 1f). In addition, there was also local m^6^A enrichment in the vicinity of stop codons. These properties are analogous to those reported for m^6^A in mammals^19^,^20^. We note that intronic CIMS are rare in the two earliest timepoints, and recorded in only 7–8 genes in timepoints 1–2. This may be expected as these temporal windows mostly sample the maternal transcriptome. Overall, we may not expect much m^6^A to be mapped in the miCLIP procedure since it incorporates poly-A selection. However, more intronic CIMS were observed following zygotic transcription, with 71–150 genes containing intronic CIMS in timepoints 3–6 (Supplementary Data 3). We return to this point later.

We generated ∼50 million reads of mRNA-seq data from each embryo timepoint used for miCLIP (Supplementary Data 1). The mRNA-seq and miCLIP data were generally correlated (Fig. 1g and Supplementary Fig. 4). We analysed those genes bearing CIMS calls and >2-fold enrichment in miCLIP reads over mRNA-seq. In libraries that represent zygotic transcription (T3–T6), we observed enrichment of Gene Ontology (GO) terms for sequence specific transcription factors (Supplementary Data 5); this was not seen in maternal libraries (T1–T2). Figure 1h illustrates *Ubx* as a TF-encoding locus with preferential miCLIP reads relative to mRNA-seq data within its 5′ UTR, and this is associated with a dense cluster of CIMS calls in the zygotic libraries. These data suggest some genes may be preferentially targeted by m^6^A, including regulatory factors.

### Characterization of *Drosophila* MTC and YTH factors

**Figure 2:**
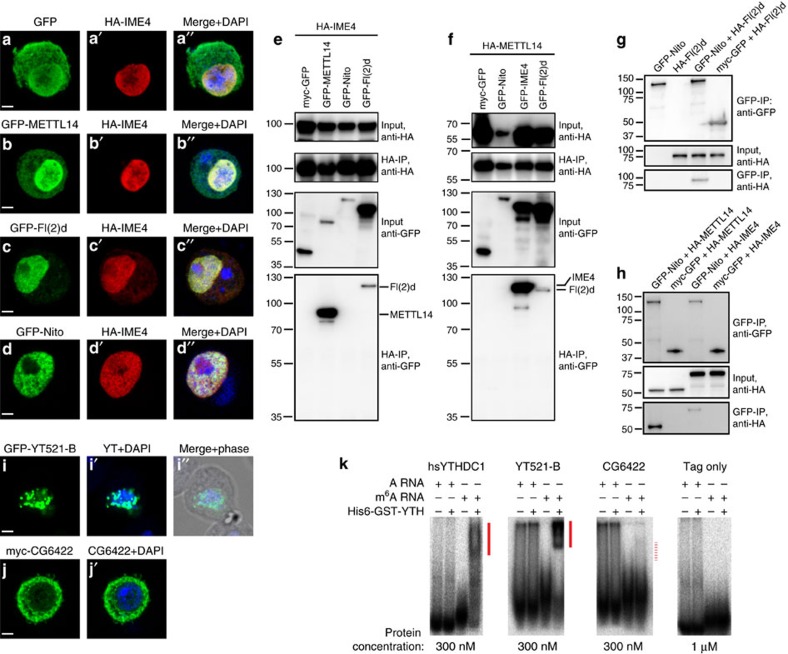
Biochemical characterization of m^6^A pathway factors in *Drosophila*. (**a**–**d**) Subcellular localization of fly homologues of mammalian factors shown to be components of the m^6^A methyltransferase complex (MTC). Tagged constructs of IME4, METTL14, Fl(2)d, and Nito were transfected into S2 cells, and all were predominantly nuclear. Scale bars, 2 μm. (**e**) Co-immunoprecipitation (co-IP) tests of MTC factors using IME4-IP (**e**) or METTL14-IP (**f**), and blotting for associated proteins. Reciprocal co-IP tests show robust association of IME4 and METTL14, whereas both interact modestly with Fl(2)d. Nito was not appreciably co-IPed with IME4 or METTl14. (**g**) Nito can specifically co-IP Fl(2)d. (**h**) Nito can specifically co-IP METTL14 and modestly co-IP IME4. (**i**,**j**) Subcellular localization of fly YTH factors, tested as tagged constructs transfected into S2 cells. YT521-B accumulates in nuclear puncta, while CG6422 is mostly cytoplasmic. Scale bars, 2 μm. (**k**) Electrophoretic mobility shift assays test the binding of purified YTH domains from the indicated factors to radiolabeled probes bearing A or m^6^A. Human YTHDC1 was used as a control, and robustly generates shifted complexes only with the m^6^A-containing probe. YT521-B similarly generates a strong shift only with the m^6^A probe. CG6422 exhibits a modest, but specific, association to m^6^A. No shifted complexes are observed with elevated concentrations of control tag-only proteins.

The *Drosophila* genome encodes single orthologs of m^6^A methyltransferase domain proteins, METTL3 (CG5933/IME4) and METTL14 (CG7818), which form the core of the methyltransferase complex (MTC)^12^,^13^,^14^. *Drosophila* IME4 and METTL14 tagged constructs were nuclearly localized in S2 cells (Fig. 2a,b). Moreover, *Drosophila* orthologs of other mammalian MTC factors, including Fl(2)d and Spenito (Nito), also exhibit nuclear localization (Fig. 2c,d).

To characterization their interactions within the MTC, we performed co-immunoprecipitation (co-IP) assays using HA- and GFP-tagged constructs. As expected, IME4 and METTL14 exhibited robust and reciprocal interactions (Fig. 2e,f). We also observed that Fl(2)d was co-IPed with both IME4 and METTL14, although this was more modest than the IME4–METTL14 interaction (Fig. 2e,f). We did not obtain appreciable pulldown of Nito with either IME4 or METTL14 (Fig. 2e,f). However, Nito could effectively pulldown Fl(2)d (Fig. 2g), and Nito immunoprecipitates yielded specific signals for METTL14 and modest detection of IME4 (Fig. 2h). Overall, these data support that Fl(2)d and Nito are components of the *Drosophila* MTC, but likely do not exclusively reside in this complex. Consistent with this view, Fl(2)d has also been co-IPed with other splicing factors and even transcription and translation factors^38^,^39^. Since mammalian Nito was recently shown to participate in the MTC^11^, we assayed *nito*[1] mutant larvae^34^ and observed a ∼30% decrease in the accumulation of m^6^A (Supplementary Fig. 7C). This might be an underestimate due to perdurance of maternal contributions of this lethal factor, and/or incomplete depletion of rRNA (whose m^6^A (Fig. 1a,b) is independent of METTL factors), but provides evidences that *Drosophila* Nito is involved in m^6^A accumulation via the MTC.

Regarding candidate readers, YTH domains are known to recognize m^6^A-modified RNA^19^,^40^. Two *Drosophila* proteins bear YTH domains, CG12076 (YT521-B) and CG6422. They display significant homology with human YTHDC1 and YTHDF1, respectively, and conserve critical residues that bind m^6^A-containing RNA^40^,^41^ (Supplementary Fig. 5). YTHDC1 is the only primarily nuclear factor out of the five mammalian YTH proteins, while the others are mostly cytoplasmic^42^. Consistent with their homologies, tagged YT521-B was nuclear while CG6422 was predominantly cytoplasmic in S2 cells (Fig. 2i,j).

To test the function of fly YTH domains, we employed gel shift assays. As a control, the YTH domain of human YTHDC1 does not shift adenosine-containing RNA, but specifically generates retarded complexes with m^6^A RNA (Fig. 2k). Similarly, the YTH domain of YT521-B specifically and robustly recognizes m^6^A-containing RNA, but not unmodified RNA (Fig. 2k). Tests of the YTH domain from CG6422 using a similar protein concentration revealed only slight binding in this assay (Fig. 2k). Nevertheless, this modest activity appeared specific, as CG6422-YTH did not generate retarded complexes with unmodified RNA, and no shift of m^6^A RNA was generated with control protein tags. These analyses support both *Drosophila* YTH proteins as functionally relevant to m^6^A, with YT521-B being particularly robust as an m^6^A-specific binding factor.

### A genetic toolkit for the *Drosophila* m^6^A pathway

**Figure 3:**
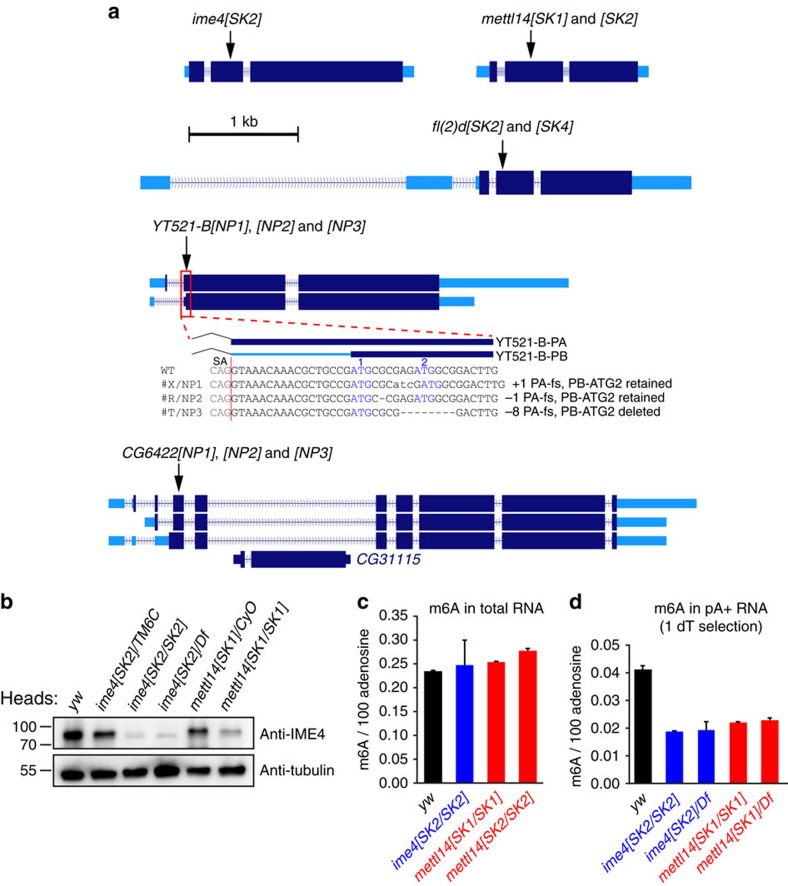
Generation and characterization of *Drosophila* m^6^A pathway mutants. (**a**) The untranslated regions of these loci are in light blue, and coding regions in dark blue. The positions of frameshift indels recovered by CRISPR/Cas9-mediated mutagenesis are indicated. Details of the mutations and predicted effects on encoded proteins are shown in Supplementary Fig. 6. Note that for *YT521-B*, alleles *[NP1]* and *[NP2]* are out of frame for YT521-B-PA and the first ATG of YT521-B-PB, but potentially can initiate in-frame at a neighboring ATG, whereas allele *[NP3]* is out of frame for the first ATG and deletes the second ATG. (**b**) IME4 Western blotting of adult heads shows that compared to *yw* control, it is reduced in heterozygotes (*ime4[SK2]/TM6C*) but nearly eliminated in homozygotes (*ime4[SK2/SK2]* and hemizygotes *ime4[SK2]/Df*). IME4 is also strongly reduced in *mettl14* homozygotes compared to heterozygotes. (**c**,**d**) Quantifications of m^6^A relative to adenosine in adult flies. m^6^A levels are not affected in the total RNA of any mutants (**c**), but are lower in *ime4* and *mettl14* mutant transcripts subjected to a single round of polyA purification (**d**). Similar results were recorded in homozygous and hemizygous mutants. Each data point represents an average of two biological replicates with two technical replicates, with the error bars representing deviation from the mean.

We used the Cas9/gRNA system^43^ to mutagenize *ime4*, *mettl14*, *fl(2)d*, *YT521-B* and *CG6422* (Fig. 3a). We selected early frameshift alleles that are expected to disrupt translation of functional domains (Supplementary Fig. 6). We highlight the utility of CRISPR in recovering diverse *YT521-B* alleles. *YT521-B* has alternative promoters and multiple potential start codons. Among our mutants, *YT521-B[NP1]* and *[NP2]* potentially retain a downstream open reading frame of YT521-B-PB, while *[NP3]* is predicted to disable translation of all YT521-B-PA and -PB isoforms (Fig. 3a).

Several *Drosophila* splicing factors whose mammalian orthologs were later recognized as MTC components (that is, Fl(2)d, Virilizer and Nito) are homozygous lethal. Consistent with this, our *fl(2)d* mutants are lethal in both sexes, thus validating the CRISPR approach to generate strong or null alleles. In contrast, all of the methyltransferase (writer) and YTH (reader) mutants were homozygous viable. A previous study of *ime4* reported that homozygous mutants were larval/pupal lethal^31^. We assess homozygous and hemizygous *ime4[SK2]* mutants by western blotting, and both revealed strong loss of IME4 (Fig. 3b). Thus, *ime4* may not be an essential gene. Moreover, we observe decreased IME4 in viable *mettl14* mutants, consistent with the notion that METTL14 stabilizes IME4 (Fig. 3b). Therefore, overall phenotypes of factors dedicated to the m^6^A pathway (that is, METTL and YTH factors) appear less severe than for general splicing factors that participate in the MTC.

We measured levels of adenosine methylation in *ime4* and *mettl14* adult females, compared to the *yw* strain used for mutagenesis. We used internal standards to assess absolute levels of methylated adenosine (Supplementary Fig. 7A,B). When analysing total RNAs, we did not detect any m^6^A_m_ (Supplementary Fig. 7D), consistent with previous reports that *Drosophila* lacks m^6^A_m_, unlike vertebrates^44^,^45^. In contrast, we detected relatively abundant m^6^A in total RNA, but there were no differences in m^6^A levels between wildtype and mutants (Fig. 3c). This was potentially due to adenosine methylation in abundant rRNA. After a single polyA selection, we find >50% reduction of m^6^A in *ime4* and *mettl14* mutants relative to *yw*, confirming they are loss-of-function for m^6^A accumulation (Fig. 3d). The frequency of m^6^A was lower following polyA selection, suggesting a minority of total m^6^A exists in mRNA. This is consistent with the presence of m^6^A in rRNA^1^,^46^ and the fact that other methyltransferases can mediate m^6^A in rRNA and snRNA^9^,^47^. We did not observe further reduction when analysing m^6^A in mutants in trans to deficiencies, suggesting they are strong alleles (Fig. 3d). Persisting rRNA following a single polyA selection may contribute to detected m^6^A (Fig. 1a,b); nevertheless, these tests validate our MTC mutants impair m^6^A accumulation.

### Behavioural and ovary defects in m^6^A pathway mutants

Although mutants in METTL and YTH factors were viable, they display several phenotypes that highlight biological settings that are particularly reliant on m^6^A-mediated regulation. We focused on defects that correlate with tissue-biased expression of m^6^A factors (Supplementary Fig. 8). Among tissues analysed in the modENCODE project^48^, expression of MTC and YTH factors is the highest or nearly highest in larval CNS, with pupal CNS and adult heads also ranking highly (Supplementary Fig. 8). Adult gonads, especially ovaries, are another location of prominent expression of m^6^A-related factors.

**Figure 4:**
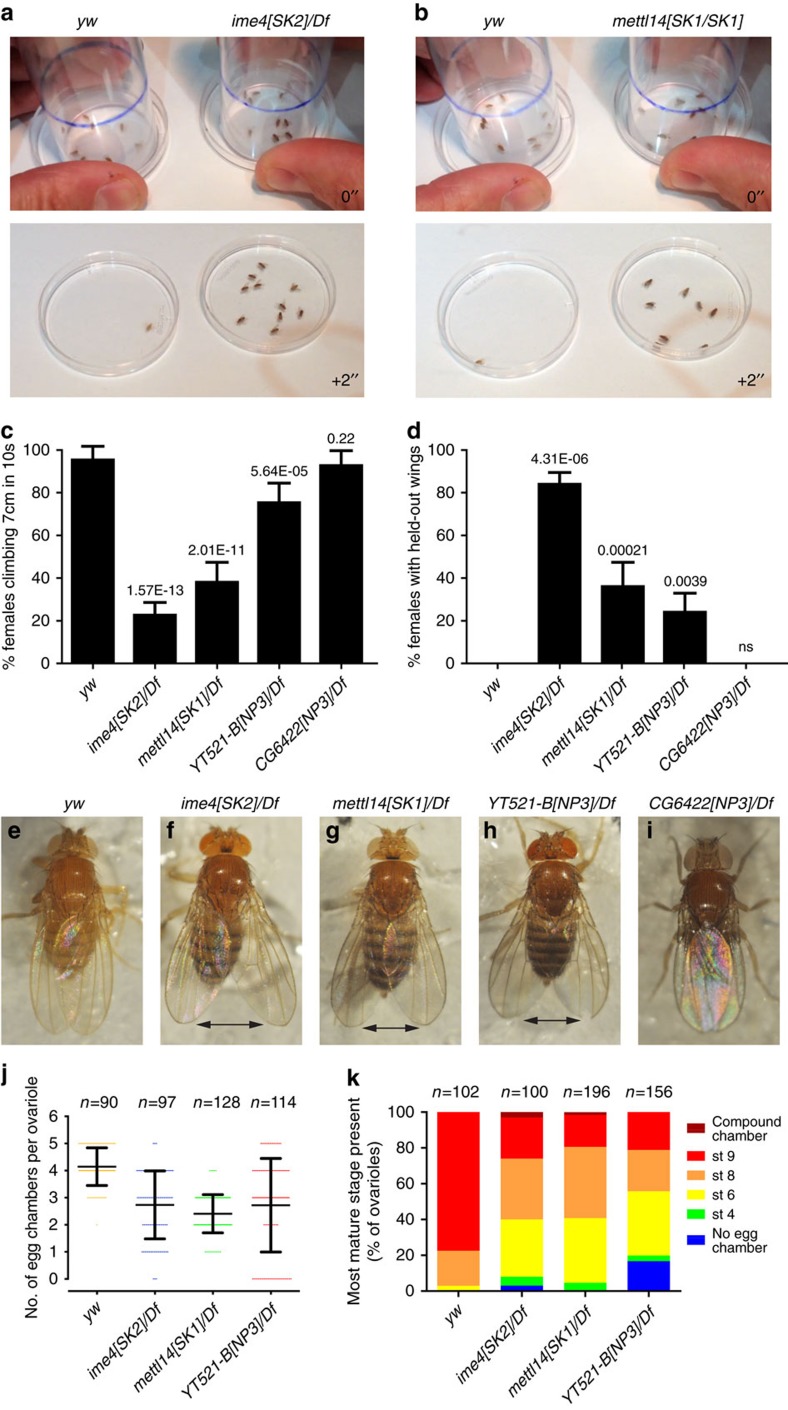
Behavioural defects in m^6^A pathway mutants. Adult female flies were analysed in these figures. (**a**,**b**) Flight defects. 10 flies were tapped down into a petri plate and their movement once the arena was opened was observed; selected video stills are shown. Most control *yw* flies jump up and/or fly away immediately, or within a couple of seconds. In contrast, *ime4* (**a**) and *mettl14* (**b**) mutants mostly remain in the plate. (**c**) Negative geotaxis assay. A total of 10 flies were placed in an empty vial and tapped to the bottom, and their ability to climb was quantified. Five independent cohorts of flies per genotype were assays, and the assay was done in triplicate for each group of flies. Nearly all control (*yw*) flies cross a 7 cm mark within 10 s; indeed, nearly all of these reached this mark in <5 s. Most *ime4* hemizygotes stayed at the bottom of the vial, and a minority slowly climb to the designated height. *mettl14* and *YT521-B* homozygous and/or hemizygous mutants also display strongly reduced negative geotaxis, whereas *CG6422* mutants were not affected. Similar behavioural defects were observed in male mutants (see Supplementary Fig. 9). (**d**) Wing postures of adult flies. The frequency of flies that exhibit held-out wings, and are unable to maintain them in a normal folded position across the back. Statistics in (**c**,**d**) are from one-way ANOVA, Tukey's Multiple Comparison Test analysis. (**e**) *yw* adult fly illustrates the normal folded position, while *ime4* (**f**), *mettl14* (**g**) and *YT521-B* (**h**) hemizygotes all show mildly held-out wings (as marked by the double arrows); *CG6422* mutant (**i**) is unaffected. (**j**) Quantification of egg-chamber number shows that different m^6^A pathway mutant ovaries exhibit fewer than in *yw* control. (**k**) Distribution of egg chamber stages shows a skew towards early stages in m^6^A pathway mutants relative to *yw* control.

Homozygous and hemizygous mutants of methyltransferase factors *ime4* and *mettl14* exhibit adult behavioural defects. These mutants are unable to fly, when provided access to an open arena (Fig. 4a,b) and are strongly compromised for negative geotaxis, as measured in their proclivity to climb after being tapped to the bottom of a vial (Fig. 4c). We observed behavioural defects in both sexes (Supplementary Fig. 9). We also noticed that instead of keeping their wings in a normally folded position, both MTC mutants exhibit mildly held-out wings (Fig. 4d). Examples of control and MTC mutant wing postures are shown in Fig. 4e–g. Some known mutants (for example, *held out wings* and *eagle*) exhibit wing positioning and flight defects, suggesting these phenotypes may be correlated.

Both m^6^A readers are well-expressed in the CNS (Supplementary Fig. 8), and therefore positioned to mediate m^6^A behavioural programs. Interestingly, *YT521-B* mutants formed an apparent allelic series. Homozygotes of the predicted weak allele *YT521-B[NP1]* fly normally, while *YT521-B[NP2]* can fly short distances when pushed or tapped. On the other hand, *YT521-B[NP3]* homozygotes and *YT521-B[NP3]/Df* hemizygotes are flightless. In addition, *YT521-B[NP3]* mutants climb poorly and frequently exhibit held-out wings (Fig. 4c,d,h). In contrast, *CG6422* mutants exhibited normal negative geotaxis (Fig. 4c) and wing positioning (Fig. 4d,i). These data indicate YT521-B is an effector of m^6^A-mediated regulation of behaviour.

We also examined m^6^A pathway mutant ovaries. Although *ime4* mutants generated by P-element excision were previously reported to have strong ovarian defects^31^, our m^6^A pathway mutants mostly exhibit normal egg chambers. Each ovariole consists of an anterior germarium that houses somatic and germline stem cells and coordinates initial stages of oogenesis, followed by a string of maturing egg chambers. Quantification of egg chamber development using anti-Vasa and anti-Hts antibodies, which mark germ cells and follicle cells, respectively (Supplementary Fig. 10) revealed very occasional compound egg chambers with >16 sister cells in *ime4* hemizygotes, as described in ref. 31. However, these were infrequent (3.1% ovarioles), were even rarer in *mettl14* hemizygotes (1.5% ovarioles), and were not observed in other m^6^A pathway mutants (Supplementary Fig. 10).

We observed modest effects on egg chamber number and stage. In our dissection conditions, most control *yw* ovarioles bore 4–5 egg chambers up to stage 9 (Supplementary Fig. 10A), among which 20% ovarioles reached stage 8 and 77% had reached stage 9 or above (Fig. 4j,k). By contrast, both *ime4[SK2]/Df* and *mettl14[SK1]/Df* genotypes exhibited 2–4 egg chambers to stage 9 (Fig. 4j). The presence of clustered basal stalk regions at the terminus of many mutant ovarioles confirmed they exhibit abbreviated egg chamber development, and were sometimes missing intermediate stage egg chambers (Supplementary Fig. 10B–D). Quantification showed that both mutants were biased to exhibit chambers of earlier stages than normal (Fig. 4k).

We also observed ovariole defects in *YT521-B[NP3]* hemizygotes (Supplementary Fig. 10E). While there is a similar trend towards fewer numbers of egg chambers (Fig. 4j), this in part due to a class of ovarioles without germ cells and egg chambers (Supplementary Fig. 10F). This phenotype was seen at a low frequency in *ime4* hemizygotes, but not in *mettl14* hemizygotes (Fig. 4k). Altogether, these analyses suggest the m^6^A pathway, functioning primarily through the YT521-B reader, regulates common processes in the nervous system and ovary, sites that exhibit elevated expression of m^6^A pathway factors.

### m^6^A pathway-specific mutants maintain Sxl in the ovary

Previous studies of splicing factors that were retrospectively recognized to be part of the MTC (for example, Fl(2)d, Virilizer and Nito) showed that they exhibit common ovarian phenotypes^34^,^35^,^36^,^37^. In particular, special viable hypomorphic alleles, or animals with ovary-specific depletions, exhibit ovarian tumours composed of germline stem-like cells that are unable to differentiate properly into cystoblasts^49^. These tumours are due to loss of Sex-lethal (Sxl), an auto-regulatory splicing factor that is the master determinant for female identity^49^. By contrast, Sxl protein is neither expressed nor required in males. Loss of Sxl, or any factor required for Sxl processing, blocks the progression of female germline stem cells (GSCs) due to deregulation of the male-specific expression program^50^. Sxl is alternatively spliced in a sex-specific manner: males include an internal *Sxl* exon that disrupts its reading frame, while this exon is skipped in female cells^49^.

**Figure 5:**
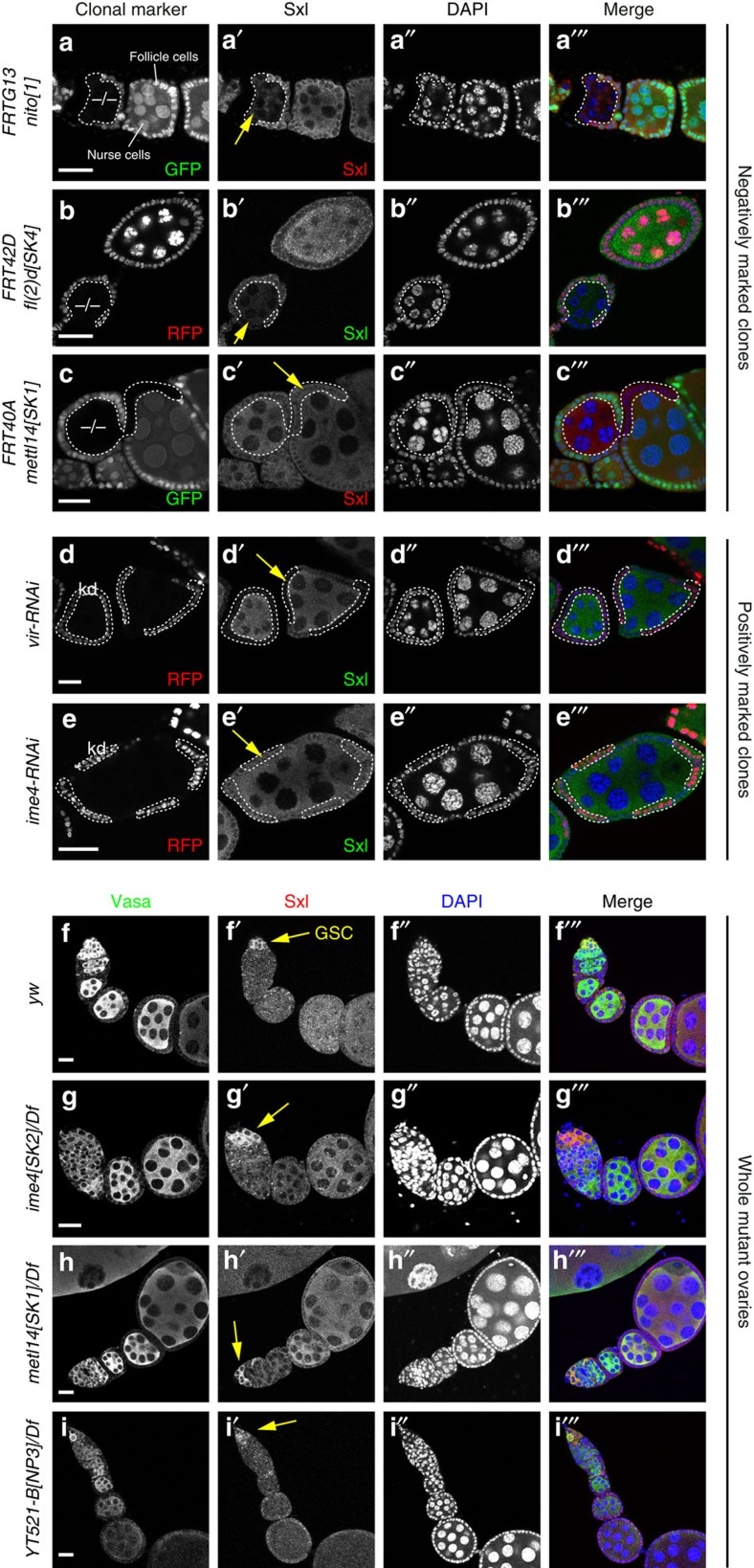
Selective requirement for classical, but not core, m^6^A factors for Sxl accumulation in ovaries. Shown are midstage egg chambers that permit comparison of somatic (follicle cells) and germline (nurse cells and/or oocyte) expression of Sxl protein. (**a**–**c**) Negatively marked mitotic clones, for which homozygous mutant cells lack either GFP or RFP (dotted clones). Sxl protein is clearly reduced in *nito*[1] (a') and *fl(2)d[SK4]* (b') clones (yellow arrows), but is maintained in *mettl14[SK1]* clones (c′). (**d**,**e**) Positively marked knockdown clones generated in somatic cells. Cells expressing the indicated RNAi transgene are labelled by RFP. Sxl protein is clearly reduced in *vir-RNAi* clones (d′) but not in *ime4-RNAi* clones (e′). (**f**–**i**) Control and mutant ovaries stained for Sxl and the germline marker Vasa. In these non-clonal settings, it is difficult to discern changes in marker protein levels as with clonal experiments. Therefore, these images include the germarium, where elevated levels of Sxl are evident in germline stem cells (GSCs). Overall normal staining patterns of Sxl and Vasa in *yw* control (**f**) are maintained in *ime4* (**g**), *mettl14* (**h**) and *YT521-B* (**i**) hemizygous ovaries. Scale bars, 20 μm.

We further confirmed the requirements of m^6^A factors that are known to affect ovarian Sxl accumulation, and compared them to METTL and YTH mutants. We evaluated mitotic clones of the published *nito*[1] allele, our new *fl(2)d[SK4]* allele, and of *mettl14[SK1]*, and observed only the former two mutants exhibited clear loss of Sxl in both germline and somatic clones (Fig. 5a–c). We also compared RNAi clones of *virilizer* and *ime4*, and observed loss of Sxl protein in the former but not the latter (Fig. 5d,e). Finally, we examined homozygous *ime4[SK2]*, *YT521-B[NP3]* and *mettl14[SK1]* mutants. While it is more challenging to evaluate potential differences in Sxl staining in non-clonal situations, we observed that all of the m^6^A pathway-specific factors maintain Sxl and also exhibit the characteristic upregulation of Sxl protein in germline stem cells of the germarium^51^ (Fig. 5f–i).

Overall, these analyses indicate different requirements of general splicing factors in the m^6^A-pathway (Nito/Fl(2)d/Virilizer) that maintain female-specific Sxl processing compared to m^6^A-pathway specific factors (*Ime4/Mettl14/YT521-B*), even though some of these factors coexist in the methyltransferase complex.

### The m^6^A pathway facilitates sex determination via *Sxl*

Besides ovarian germline differentiation, *Sxl* regulators are important in the soma to establish sex-specific Sxl expression and processing. In the early embryo, the *Sxl[Pe]* promoter is activated by the Da/Sc bHLH heterodimeric complex, followed by a shift of *Sxl* transcription from the *Sxl[Pm]* promoter and locking-in of sex-specific splicing patterns via positive autoregulation by Sxl (ref. 49). The establishment of Sxl auto-activation in female embryos is independent of maternally deposited Sxl, and thus needs to be achieved *de novo*^52^.

In this context, defects in establishing Sxl autoregulation are manifest in sex imbalance of progeny, wherein insufficient Sxl function results in female-specific lethality. We did not observe female depletion in the progeny of homozygous or hemizygous crosses for any of our m^6^A factors (Supplementary Fig. 11), indicating that sufficient Sxl auto-activation occurs in our mutants. Nevertheless, we investigated the consequences of pushing this system by reducing the dosage of *Sxl* or *Sxl*-regulating factors. These tests are performed by combining alleles of candidate factors in the mother, and crossing to null *Sxl[7BO]/Y* fathers (as Sxl is not required in males). It is relatively rare for individual maternal mutations to dominantly enhance the inviability of *Sxl/+* females (two of these being *daughterless* (*da*)^53^ and the special antimorphic allele *fl(2)d[D1]*^54^), but additional factors can enhance *Sxl/+* in homozygous state, or when present as compound heterozygotes with another sensitizing mutation in the *Sxl* regulatory machinery^54^,^55^,^56^.

**Figure 6:**
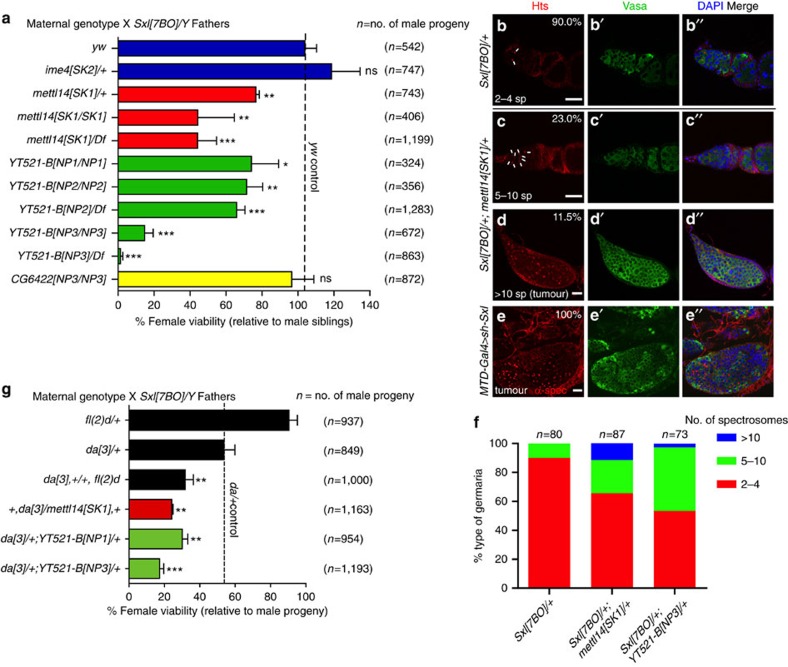
The m^6^A pathway promotes female sex determination. (**a**) Genetic interactions of m^6^A pathway mutants with *Sxl*. The indicated maternal genotypes were crossed with *Sxl* null fathers, and the viability of female adults relative to sibling male adults was quantified. *mettl14* mutants exhibit substantial loss of female *Sxl* heterozygotes, while *YT521-B* mutants, especially of the strong allele *YT521-B[NP3]*, exhibit profound loss of females that lack a copy of *Sxl*. (**b**–**e**) Germaria regions of ovarioles stained for spectrosome markers in red (Hts, (**b**–**d**) or alpha-spectrin, **e**), germline marker in green (Vasa, b′–e′), merged panels with DAPI (b″–e″). Scale bars, 20 μm. (**b**) Nearly all *Sxl/+* ovarioles exhibit the normal 2–4 spectrosomes (sp, marked by white lines). (**c**) Nearly one fourth of *Sxl/+; mettl14/+* ovarioles exhibit 5–10 spectrosomes, while >11% exhibit ovarioles full of undifferentiated germline stem cell-like tumours (**d**). (**e**) The latter resemble tumorous ovaries that result from germline knockdown of *Sxl*, although these are obtained with full penetrance. (**f**) Quantification of supernumerary spectrosome phenotypes in *Sxl* dominant interaction tests with m^6^A mutants. (**g**) Genetic interactions of m^6^A pathway mutants with *Sxl* and *da*. *Daughterless* (*da*) exhibits haploinsufficiency in *Sxl/+* females, and thus serves as a genetically sensitized background. Thus, loss of one allele of *fl(2)d* has little effect on *Sxl/+*, but concomitant heterozygosity of *da* and *fl(2)d* enhances female lethality of *Sxl/+*. Similarly, in the *da* sensitized background, heterozygosity for *mettl14* and different *YT521-B* alleles all dominantly enhance female loss in *Sxl/+*.

We first tested the effect of m^6^A pathway mutations in *Sxl* heterozygotes. Because of near infertility of *ime4[SK2]* homozygotes, we examined them as heterozygotes, and these exhibited normal viability of *Sxl/+* females. This indicated that females could tolerate *Sxl* heterozygosity even in some sensitized states. However, *mettl14* heterozygotes exhibited mild reduction of *Sxl/+* females, and this effect was substantially stronger in homozygous condition (44% of expected females). We confirmed these results in trans to a deficiency (Fig. 6a), clearly implicating METTL14 in Sxl regulation.

Among YTH factors, *CG6422* mutants did not enhance *Sxl*, but the consequences of YT521-B loss were striking. Homozygotes of *YT521-B[NP1]* and *[NP2]* which we predict as retaining some translation, exhibit modest enhancement of *Sxl/+* (74% and 71% female survival, respectively). However, homozygotes of *YT521-B[NP3]*, which we predict to disrupt both isoforms, exhibited profound sex bias with only 16% of *Sxl/+* females surviving to adulthood (Fig. 6a). Moreover, *YT521-B[NP3]/Df* hemizygotes recapitulated nearly complete inviability of *Sxl/+* females. These assays demonstrate YT521-B as the major m^6^A effector during sex determination, and implicate both isoforms in the control of female-specific Sxl splicing.

Further impact of this genetic sensitization was apparent in adult female survivors. *Sxl/+* exhibits relatively normal oogenesis, including 2–4 round spectrosomes that are characteristic of GSCs and cystoblasts, and are labelled by Hts or alpha-spectrin antibodies (Fig. 6b). However, a substantial population of surviving females that were doubly heterozygous for *Sxl/+; mettl14/+* or *Sxl/+; YT521-B* exhibited ovarioles with excess spectrosomes that could be classified as mild (5–10, Fig. 6c) or tumorous (>10, Fig. 6d). These phenotypes resemble the block in GSC differentiation documented for loss of *Sxl* in the female germline, as shown with *Sxl* knockdown using *MTD-Gal4* (Fig. 6e). We quantified spectrosome numbers in dominant genetic interaction tests of *Sxl* with *mettl14* and *YT521-B* (Fig. 6f). These data from different developmental contexts support the view that m^6^A factors facilitate Sxl function.

Finally, we performed additional genetic sensitization of the *Sxl* background by removing an allele of maternal *daughterless* (*da*), which further compromises Sxl autoregulation. In our tests, we crossed *Sxl* fathers to a maternal genetic background containing *da*[3], resulting in about half the expected survival of females (Fig. 6g). This background can help reveal functions of loci that are sufficient even in *Sxl/+* backgrounds. For example, while *Sxl/+; fl(2)d/+* exhibits normal female survival, further reduction of *da* causes synergistic loss of female viability (Fig. 6g). With this framework in hand, we observed that *mettl14* dominantly enhanced *Sxl/+; da/+* female lethality. In this sensitized background, *CG6422[NP3]/+* resulted in female survival of 19.2%. However, as tests of *CG6422[NP2]/+* and *CG6422[NP1]/+* revealed no deviation from *Sxl/+; da/+*, the role of this reader remains equivocal (Supplementary Fig. 11). On the other hand, heterozygosity for multiple *YT521-B* alleles strongly enhanced *Sxl/+; da/+* female lethality, with relative strengths that paralleled the *Sxl/+* interaction tests (Fig. 6g). These data indicate *mettl14* and *YT521-B* function in combination with maternally supplied *Da* for *Sxl* activation in females. Altogether, these assays link multiple core m^6^A pathway components to the female sex determination cascade via Sxl.

### The m^6^A pathway regulates female-specific splicing of *Sxl*

**Figure 7:**
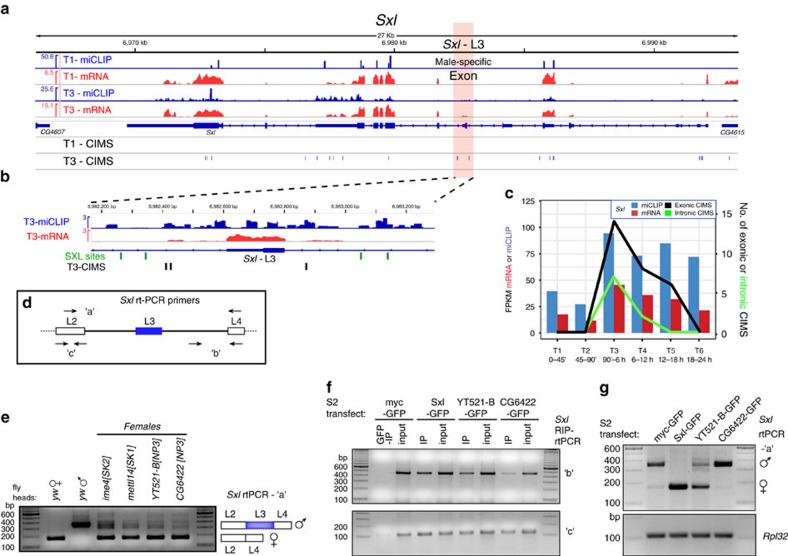
The m^6^A pathway facilitates female-specific *Sxl* splicing. (**a**) RNA-seq, miCLIP, and CIMS/m^6^A calls at the *Sxl* locus. Genome browser screenshot shows that *Sxl* transcript is detected at relatively comparable levels in maternal RNA (T1, 0–45' embryos) and in embryos following zygotic activation (T3, 90'–6 h embryos). However, there is comparably less miCLIP signal and no CIMS calls in the T1 library, whereas abundant miCLIP signal and CIMS calls were found in the T3 library. (**b**) Enlarged region centred on the L3 male-specific Sxl-exon (in pink, **a**) highlights intronic miCLIP signal, three intronic CIMS calls, and two pairs of Sxl autoregulatory binding sites that flank the alternatively spliced exon. (**c**) Analysis of RNA-seq and miCLIP expression (left Y-axis), and exonic/intron CIMS sites (right *Y*-axis) across embryogenesis emphasizes that miCLIP and CIMS calls at Sxl are found specifically in T3. (**d**) Summary of *Sxl* rt-PCR primers. (**e**) rt-PCR analysis of head transcripts shows the segregated accumulation of female and male isoforms in each sex, whereas m^6^A writer and reader mutant females detectably accumulate the male isoform. (**f**) RIP-rtPCR assays of GFP-tagged constructs transfected into (male) S2 cells, assayed for *Sxl* amplicons ‘b’ and ‘c’ (**d**). As with Sxl, YT521-B and CG6422 associate with *Sxl* transcripts. (**g**) Effect of transfected constructs on Sxl splicing in S2 cells. As with ectopic Sxl, overexpression of YT521-B induces a switch towards the female-specific *Sxl* splicing pattern.

By themselves, the genetic interaction data do not imply any particular aspect of the Sxl cascade as being regulated by m^6^A. However, inspection of the miCLIP data points to an intriguing possibility that *Sxl* is a direct target. *Sxl* exhibits substantial miCLIP reads across its exons in multiple libraries, but CIMS calls appeared specifically during timepoint 3 (1.5–6 h) (Fig. 7a,b). This was notable since there is substantial maternal deposition of *Sxl*, and transcript level at the time of zygotic transcription only increases its level a few fold (Fig. 7c). More provocative was the temporally regulated presence of miCLIP reads and CIMS calls in *Sxl* introns (Fig. 7a,b). Although intronic signals were modest, as expected given that miCLIP utilizes poly-A selection, *Sxl* exhibited the largest number (7) of intronic CIMS calls in timepoint 3, and these CIMS sites were supported by the third highest number of intronic miCLIP reads for any genes in this library (Supplementary Data 6). Of note, timepoint 3 includes the stage during which activation of the Sxl auto-regulatory splicing circuit in females must occur^49^. In fact, even when considering all 6 miCLIP datasets, no gene generated as many intronic CIMS calls as *Sxl*. Furthermore, three of these CIMS calls reside in the two introns that flank the sex-specifically spliced *Sxl* exon (Fig. 7b), close to the locations of Sxl autoregulatory poly(U) binding sites^57^.

Subsequent to this stage, even though *Sxl* transcript levels remain fairly constant during embryogenesis, *Sxl* intronic CIMS calls declined substantially in timepoint 4 and were not detected in the last two libraries (Fig. 7c). Incidentally, *fl(2)d* is another locus with quite prominent intronic CIMS calls (Supplementary Fig. 12), and ranks in the top three of all genes in timepoints 3, 4 and 5, both in terms of the number of intronic CIMS sites and in CIMS reads (Supplementary Data 6). Indeed, besides *Sxl* and *fl(2)d*, only three other genes exhibited four or more intronic CIMS sites in any of the 6 miCLIP libraries (Supplementary Data 6).

We tested whether *Sxl* splicing was altered in female mutants of m^6^A factors using primer pairs against the exons flanking the sex-specific alternatively spliced exon (Fig. 7d, amplicon ‘a’). In normal tissues, males accumulate a longer isoform as a result of inclusion of exon L3, whereas females accumulate a shorter isoform of skipping exon L3. We observed that *ime4* and *mettl14* mutant females accumulate both isoforms in heads, indicating defective sex-specific splicing of *Sxl* (Fig. 7e). Interestingly, both *YT521-B* and *CG6422* also showed accumulation of male *Sxl* products in female heads (Fig. 7e), consistent with the notion that both m^6^A readers have a role in *Sxl* regulation.

To test if the observed splicing effect might be direct, we transfected GFP-tagged Sxl and YTH constructs into (male) S2 cells, and assayed their binding to nascent and total (nascent+spliced) *Sxl* transcripts using intron-exon and exon-exon primer pairs, respectively (Fig. 7d). We observed robust detection of both amplicons in positive control Sxl-RIP tests, while control GFP-RIP tests were negative (‘b’ pair) or barely detected (‘c’ pair) (Fig. 7f). With this context, we obtained evidence that YT521-B could co-immunoprecipitate *Sxl* primary transcripts using both amplicons (Fig. 7f). CG6422-RIP modestly amplified with the intron-exon primary transcript (‘b’ pair), but generated stronger signal with the exonic amplicon (‘c’ pair), perhaps consistent with the fact that it is a cytoplasmic reader. Finally, we used these transfected materials to assess the status of *Sxl* splicing itself. Overexpression of Sxl (female) complementary DNA (cDNA) is expected to lead to preferential amplification of the shorter isoform (Fig. 7g). Interestingly, overexpression of YT521-B, but not CG6422, can switch the splicing pattern of endogenous Sxl from male to female (Fig. 7g).

Overall, while there is molecular evidence that both YTH readers are involved in *Sxl* regulation and/or function, the stronger genetic interactions of *Sxl* with *YT521-B* and the sufficiency of YT521-B to redirect *Sxl* splicing suggest it is the major effector in this process. Altogether, these data provide evidence that *Sxl* is a prominent target of m^6^A, and that this modification pathway mediates its efficient sex-specific processing and function during female determination.

## Discussion

Among major model organisms, studies of the m^6^A pathway have been notably limited in *Drosophila*^31^. We address this by providing the first single-nucleotide transcriptome maps of m^6^A modifications in flies, by biochemical demonstration of m^6^A binding by YTH factors, particularly by YT521-B, and by generation of a comprehensive set of mutants in core m^6^A pathway components. This combined genomic and genetic toolkit provides new insights into m^6^A-mediated regulation and opens the powerful *Drosophila* system for further investigation of developmental and/or tissue-specific mRNA methylation. Our work reinforces and extends recently published studies from the Roignant and Soller groups, who independently reported analysis of the *Drosophila* m^6^A pathway and its roles in sex determination and behaviour^58^,^59^. All of these studies report mutually supportive data on molecular and genetic characterization of *Drosophila* m^6^A factors, and converge on the finding of a shared pathway involving METTL and YTH factors, especially YT521-B, to facilitate the Sxl autoregulatory switch in females.

Although most splicing occurs co-transcriptionally, *Sxl* is a notable locus whose alternative splicing is uncoupled from transcription^60^. This may underlie why it was preferentially picked up in our miCLIP sequencing. It remains to be understood how m^6^A and YT521-B interface with other known *Sxl* splicing machinery. Since *Sxl* splicing requires specialized factors, including Sxl protein itself^61^, one hypothesis is that YT521-B may interact with setting-specific cofactors to mediate different regulatory outcomes. Alternatively, general factors likely come into play, and there are considerations regarding how the MTC may link to Sxl regulation. Of note, there is extensive data on physical associations amongst the m^6^A/splicing factors Fl(2)d, Nito and Vir, with Sxl, as well as with the U1 snRNP and U1 snRNP^33^,^34^,^37^,^54^. As Fl(2)d, Nito and Vir co-purify with the pre-catalytic spliceosome^62^, they might potentially serve as a bridge between the spliceosome and the m^6^A catalytic core. Does this suggest a link between writer and reader complexes? Finally, there may be potential involvement of the cytoplasmic reader *CG6422* in *Sxl* regulation. Although its impact appears subsidiary to that of YT521-B, it will be interesting to elucidate how the two readers act in relation to each other and to m^6^A.

The fact that *Drosophila* sex determination is sensitive to m^6^A pathway function seems curious, given that the mammalian m^6^A pathway is critical for dosage compensation^11^,^63^,^64^,^65^. The strategies that establish sex-specific gene regulation are very different between flies and mammals, since the former involves activation of X-linked transcription in males while the latter involves silencing of one X chromosome in females. The levels at which m^6^A function are also distinct, since our data suggest that splicing of the master sex determination factor Sxl is influenced by intronic m^6^A, while the exons of the XIST non-coding RNA that imposes X silencing are highly modified by m^6^A (ref. 11). In any case, control of sex-specific gene regulation via non-coding m^6^A is a shared feature of diverse metazoans.

Finally, we and others also defined behavioural defects associated with loss of m^6^A factors^58^,^59^. This correlates with the fact that the expression of m^6^A writer and reader factors is especially high in CNS. However, behavioural defects are observed in both sexes for m^6^A mutants indicating a more general effect. Despite neural enrichment of most m^6^A factors, this does not have to due to neural defects per se. However, Roignant and colleagues reported important evidence that *ime4* mutant behaviours were rescued by re-expression in neurons^59^, indicating cell-autonomous roles for m^6^A-mediated regulation within the nervous system.

Although there have not been many studies on the role of m^6^A writer or reader factors in the nervous system of other species, FTO, an enzyme once thought to target m^6^A, was reported to affect the activity of dopaminergic neurons^66^. However, recent studies show that FTO instead primary targets a related nucleotide, *N*^6^, 2′-*O*-methyldimethyladenosine (m^6^A_m_).^67^. As well, alternative polyadenylation that generates extended 3' UTR isoforms in the nervous system^68^ was recently correlated with enhanced m^6^A in terminal exons of mammalian brain transcripts^24^. It remains to be seen how m^6^A affects neural gene expression, but the *Drosophila* should be powerful to decipher this, as well as to place the importance of such regulation into neuroanatomical context.

## Methods

### Mapping m^6^A modifications in the transcriptome

#### mRNA-seq and miCLIP libraries

We mass-collected *Canton S* embryos across these developmental timepoints: 0–45′, 45–90′, 1.5–6 h, 6–12 h, 12–18 h and 18–24 h. In principle, zygotic transcription does not initiate until ∼2 h. However, due to variability in time of egg laying after fertilization, it can be difficult for early timepoints to be staged precisely. Therefore, we changed egg laying plates frequently when collecting the earliest timepoints, and split these into two stages that should both reflect the maternal transcriptome. Aliquots of these RNA samples were subjected to Illumina mRNA TruSeq Stranded library Prep and sequenced on HiSeq 2500, 2 × 50, High Output at the New York Genome Center (New York City).

The same RNA samples were subjected to miCLIP using the described strategy^23^. Briefly, RNA was fragmented and incubated with the anti-m^6^A antibody (Abcam ab151230). After RNA-antibody binding was complete, samples were crosslinked twice in a Stratalinker 2400 (Stratagene) using 150 mJ per cmˆ2. Then, crosslinked RNA-antibody complexes were immunoprecipitated using Protein A/G magnetic beads (Thermo). After immunoprecipitation was complete, immunoprecipitation reactions were washed using high-salt conditions that included ionic detergent to remove non-crosslinked RNA. Crosslinked RNA-antibody complexes were then radiolabeled with T4 PNK (NEB), ligated to a 3′ adaptor using T4 RNA Ligase I (NEB), and further purified using SDS–polyacrylamide gel electrophoresis (SDS–PAGE) and nitrocellulose membrane transfer. RNA fragments containing crosslinked antibody peptide were recovered from the nitrocellulose membrane using proteinase K (Invitrogen) digestion.

Recovered RNA fragments were then subjected to library preparation. First-strand cDNA synthesis was performed using SuperScript III (Life Technologies) and iCLIP-barcoded primers^69^, which contain a region complementary to the 3′ adaptor on the RNA. After cDNA synthesis was complete, cDNA was purified from excess primer using denaturing PAGE purification. Then, cDNA was circularized using CircLigase II (EpiCentre), annealed to the iCLIP Cut Oligo^69^, and digested using FastDigest BamHI (Thermo). This generated linear cDNA with ends compatible for priming for amplification. Libraries were amplified using Accuprime SuperMix I (Invitrogen) and P5 and P3 Solexa primers. Finally, libraries were purified using Agencourt AMPure XP beads (Beckman Coulter) and sequenced at the Weill Cornell Medicine Epigenomics Core using an Illumina HiSeq 2500 instrument. Libraries from the samples were sequenced together on one lane in paired-end mode, with a read length of 50 bases.

#### Read processing

Fastq files for six embryo timepoints were adaptor trimmed using Flexbar (https://github.com/seqan/flexbar): flexbar -r read1.fastq -p read2.fastq -f i1.8 –a [adaptor sequences file] --pre-trim-phred 30 -n 20 -s –t [timepoint suffix]; and demultiplexed using pyBarcodeFilter.py from the pyCRAC software suite^70^ using the command: pyBarcodeFilter.py -f [timepoint suffix]_1.fastq -r [timepoint suffix]_2.fastq –b [barcode file]. PCR duplicates in the dataset were removed using pyFastqDuplicateRemover.py script^70^, and the read headers were then altered to be compatible with the CIMS software package using the following awk command: awk -F ‘[_/]’ ‘/ˆ>/{print $1‘_’$2‘_’$3‘/’$4‘#’$3‘#’$2; getline($9); print $9}’. Paired-end RNA-seq data from control mRNA and miCLIP for six embryo developmental timepoints were mapped to the *Drosophila* reference genome sequence (version r5.45) with Subread-align package from the Subread software suite^71^. Read counts for each gene was extracted from the RNA-seq bam files using featureCounts package from the Subread software. The read counts per gene were then normalized to obtain FPKM values using DEseq package from R Bioconductor.

#### miCLIP analysis and mutation calling

The reads were mapped to the *Drosophila* reference genome sequence (version r5.45) with Novoalign (Novocraft) or the Subread-align package from the Subread software suite^71^. For the Novoalign alignment, a maximum alignment score of 85 was allowed and the parameters were adjusted to map short reads. For alignment using the subread-align, the alignment was performed using default parameters for mapping paired-end RNA sequence data. Positions of C→T transitions from the mapped data were identified using the CIMS software package^72^. To filter background mutations, the coordinates identified to be C→T transitions from the CIMS package were cross-referenced against the *Drosophila* SNP database (*Drosophila* Genetic Reference Panel)^73^. For each mismatch position, the CIMS software identifies the unique tags that span the mismatch position (*k*) and the number of mutations that support the mismatch (*m*). However, the software does not provide a false discovery rate for the mismatches identified. Therefore, to minimize the signal-to-noise in identifying C→T transitions, we only considered sites with an *m*/*k* ratio 1–50%. The number of CIMS identified from each timepoint during embryonic development is provided in Supplementary Data 2.

#### Peak calling from unique clustered tags

The overlapping unique aligned reads were clustered to identify peaks using the tag2cluster.pl script from the CIMS software package. The peak clusters were then filtered for alignment with at least four stacked reads to obtain the final peak coordinates. The numbers and the genomic coordinates of the peaks from different timepoints during embryonic development are provided in Supplementary Data 3 and 4.

#### Annotation of CIMS sites

The C→T transitions identified from each timepoint was assessed for sites with Adenosine nucleotide preceding the mismatch site. We filtered such sites to obtain the final list of CIMS sites and performed annotation using the Peak Annotation and Visualization software (PAVIS)^74^. The detailed annotation of CIMS sites from different timepoints is provided in the Supplementary Data 4.

#### Motif analysis

Motif analysis on filtered CIMS sites with Adenosine nucleotide preceding C to T transitions was performed using Seqlogo software^75^. For this analysis, the filtered CIMS were subdivided into sites with high (*m*>10), medium (5≤*m*≥10) and low (*m*<5) mismatch values. Next, FASTA input files with ten nucleotides upstream and downstream of the mutation site was obtained using bedtools v.2.25.0 from the *Drosophila* reference genome sequence (version r5.45). Seqlogo was generated with the CIMS centred on the motif using the options -l and -m to specify the nucleotide ranges in the final motif.

#### Metagenes

RNA features (5′ UTR, CDS and 3′ UTR) for the Drosophila genome were obtained from the UCSC Data browser for dm3 version of the genome. Metagenes were constructed using custom shell and R scripts. Briefly, for every gene with CIMS identified during the developmental timecourse, the RNA features were first divided into 1 nt windows using bedtools makewindows tool (https://github.com/arq5x/bedtools) and coverage per nucleotide from the miCLIP data were obtained for each RNA feature. Per-nucleotide coverage was then rescaled to represent a meta-feature of 1500, bp (200 bp for 5′ UTR, 1,000 bp for CDS; and 300 bp for 3′ UTR), where each meta-feature gets a weight of 1 by normalizing the meta-feature to the coverage per transcript. The average read density per-nucleotide from meta-features of 1,500 bp, representing multiple genes were plotted as a miCLIP metagene.

#### Analysis of intronic CIMS

CIMS sites that existed unambiguously or otherwise within exons were filtered out. The remaining sites were examined for occurrence within introns. To calculate the number of intronic sites, intronic CIMS within individual isoform models (per gene) were summed, and the highest numbers were reported in Supplementary Data 6.

### Analysis of protein complexes and subcellular localizations

To generate the GFP- or HA-tagged plasmids, we cloned full-length cDNAs for IME4 (AT20169), Mettl14 (LD06016), Fl(2)d (LD21616), and CG6422 (FI18276) into the pENTR vector (Invitrogen), and transferred these into the *Drosophila* Gateway vector pAGW (N-terminal GFP fusion) or pAHW (N-terminal HA tag), respectively. YT521-B full- length sequence (encodes YT521-B-PA, 721aa) was obtained by PCR from a cDNA library and cloned into pAGW. GFP was cloned into pAWM as a control. Primers used to amplify these open reading frames are listed in Supplementary Data 7.

We used different co-IP methods that were optimized for signal detection. For tests involving HA-methyltransferase (IME4 or METTL14) pulldowns, we used the following strategy. Each well of a 6-well plate of S2 cells was transfected with 0.5 μg of HA-tagged construct and 1.5 μg of GFP-tagged construct using Effectene (Qiagen). After incubation for 3 days, cells were washed with PBS and lysed with co-IP lysis buffer (10 mM Tris–HCl, pH 7.5, 300 mM NaCl, 1 mM EDTA, 1% Triton X-100, 1 × protease inhibitor (Roche)) on ice for 30′, and then followed by two centrifugation at 20,000 *g* for 10′ at 4 °C. The cleared cell lysates are incubated with HA antibody (F-7, Santa Cruz, #Sc-7392) conjugated beads for 2 h. Beads were washed with co-IP wash buffer (10 mM Tris-HCl, pH 7.5, 500 mM NaCl, 1 mM EDTA, 1% Triton X-100) for five times and then resuspended in 30 μl 3 × sample buffer.

For tests involving GFP-Nito pulldown, cells were transfected using Effectene. After 2 days, cells were washed with PBS and lysed with lysis buffer (50 mM Tris (PH 7.4), 150 mM NaCl, 1% NP-40, 1 × protease inhibitor) on ice for 30 min, and then cleared at 20,000*g* for 10′ at 4 °C. Supernatants were transferred to pre-cooled tubes and incubated with 25 μl equilibrated GFP-Trap beads (ChromoTek) for 2 h at 4 °C. Beads were spun down and washed two times with lysis buffer and then resuspended in 2 × SDS-sample buffer.

For western blotting, we used mouse anti-HA (F-7, 1:1000, Santa Cruz, #Sc-7392), rabbit anti-GFP (1:1000, Invitrogen, #A11122), rabbit anti-c-myc (A14, 1:1000, Santa Cruz, #Sc-789), anti ß-tubulin (1:500, DSHB, #E7) and guinea pig anti-IME4 (1:10,000, gift of Terry Orr-Weaver, MIT). Secondary goat anti-mouse antibodies conjugated to HRP (Jackson ImmunoResearch #115-035-062), goat anti-rabbit antibodies conjugated to HRP (Jackson ImmunoResearch #711-035-152), and donkey anti-guinea pig antibodies conjugated to HRP (Jackson ImmunoResearch #706-035-148) were used at 1:10,000. All of the uncropped western blots shown in the main figures are provided in Supplementary Fig. 13.

For cell staining, transfected S2 cells were placed on slides treated with poly-lysine and fixed with 4% paraformaldehyde for 15′. Cells are permeabilized using 0.1% Triton in PBS (PBST) and followed by incubation with blocking buffer (1% Goat serum in PBST) for 1 h. Primary antibodies used were mouse anti-Myc (A14, 1:200, Santa Cruz, #Sc-789), mouse anti-HA (F7, 1:50, Santa Cruz, #Sc-7392), and rabbit-anti-GFP (1:1000, Invitrogen, #A11122), and secondary antibodies Alexa-568 goat anti-mouse (1:1000, Invitrogen #A-11031) and Alexa-488 anti-rabbit (1:1000, Invitrogen, #A11034). Cells were mounted in Vectashield mounting buffer with DAPI (Vector Laboratories).

### Cloning of YTH domains and protein expression

We generated alignments of YTH domains using the Endscript server^76^. Codon-optimized open reading frames from CG12076 (amino acids from 241–417, Uniprot accession number: Q9VZQ1-1) and CG6422 (amino acids from 372 to 529, Uniprot accession number: Q9VBZ5-1) were synthesized *in vitro* and cloned in pT7CFE1-Nhis-GST-CHA vector (cat. #88871, Thermo Fisher Scientific) at *Bam*HI and *Xho*I sites. His-GST-tagged proteins were expressed using mammalian cell-free protein expression system (1-step human high-yield mini IVT kit, cat. #88890, Thermo Fisher Scientific) for 6 h at 30 °C, as per the manufacturer’s instructions. Proteins were purified using MagneGST protein purification system (cat. #V8611, Promega). Proteins were eluted in 10 mM reduced glutathione in 50 mM Tris-HCl (pH 8.8). The eluted proteins were dissolved in exchange buffer (10 mM HEPES pH 7.4, 150 mM KCl, 0.01% NP40, 5% glycerol, and 1 mM DTT) following buffer exchange using a centrifugal filter (Millipore, 30 kDa cut-off). Proteins were stored at 4 °C until use.

### Electrophoretic mobility shift assay

Radiolabeled RNA probes were synthesized by *in vitro* transcription using AmpliScribe T7-Flash Transcription Kit (Epicentre) with some minor modifications. A double stranded DNA template (sense strand, 5′-ATATTAATACGACTCACTATAGTTTTTTTGGACTTTTTTTT-3′; anti-sense strand: 5′-AAAAAAAAGTCCAAAAAAACTATAGTGAGTCGTATTAATAT-3′) at a final concentration of 100 nM was incubated with 1 mM ATP or m^6^ATP, GTP and UTP, and 2 μl of ^32^P-α-CTP (3000 Ci per mmol, 10 mCi per ml), and other reaction components as per the kit’s instructions in a 20 μl reaction. The reactions were incubated at 37 °C for 30 min. RNA probes were purified using RNA Clean and Concentrator 5 kit (Zymo Research). His-GST-tagged YTH domains from CG12076 and CG6422 were mixed with heat-denatured A or m^6^A RNA probes (20,000 c.p.m.) in binding buffer (10 mM HEPES pH 7.4, 50 mM KCl, 1 mM EDTA, 0.05% Triton-X-100, 5% glycerol, 10 μg per ml salmon DNA, 1 mM DTT, 2 U per μl RNasin). The reactions were incubated at room temperature for 30 min. Free RNA and protein-RNA complexes were resolved on a 6% DNA retardation gel (Novex) at 4 °C in 0.5 × TBE for 60 min at 90 V. Gels were collected on Whatman sheets, sealed in a plastic bags, and exposed to a phosphor screen for 1 h. Autoradiograms were obtained using a phosphorimager (Typhoon, GE Amersham).

### *Drosophila* genetics and immunostaining

We used the transgenic gRNA system for CRISPR/Cas9-mediated mutagenesis to generate new mutants in m^6^A factors, using the published crossing schemes^43^. Guide RNA sequences are listed in Supplementary Data 7. We analysed 8–16 individual candidate flies and obtained multiple indel alleles in all experiments; we kept the out-of-frame mutants, as described in Fig. 3 and Supplementary Fig. 6.

To analyse hemizygous conditions, we utilized these deficiency chromosomes (Bloomington Stock Center): *Df(3R)Exel6197* (removes *ime4*), *Df(2L)BSC111* (removes *mettl14*) and *Df(3L)ED208* (removes *YT521-B*). All of these were genotyped in trans to mutants to confirm the absence of the wildtype allele. To detect genetic interactions with the Sxl system, we used *fl(2)d*[1] (Bl-36301), the deletion allele *Sxl[7BO]*. To test compound genetic interactions we crossed m^6^A-related mutants to a double balanced stock of *FRT40A da*[3]*/CyO;* (gift of Hugo Bellen) to obtain the desired maternal genotype (thus removing balancer chromosomes), and then crossed these to *Sxl[7BO]/Y* fathers. For *Sxl/+* interaction tests, we performed at least three independent crosses for each genotype, and transferred each cross at least three times. Adult progeny in each vial were sexed, and the ratio of observed female to male sibling progeny was determined to infer female lethality rate.

Ovary stainings were performed on homozygous and hemizygous mutant backgrounds, as well as in clonal settings. Mitotic clones were induced in the following genotypes: (1) *hsFLP; ubi-GFP, FRT40A/mettl14[SK1], FRT40A*; (2) *hsFLP; FRT42D hRFP/FRT42D, fl(2)d[SK4]*; (3) *hsFLP; FRTG13, nito*[1]*/FRTG13, ubi-GFP*. RNAi clones were induced in the progeny of *hsFLP;; act>CD2>Gal4, UAS-ubi-RFP* X *UAS-shRNA*; using the following TRiP knockdown lines (Bloomington Stock Center): *nito* (BL-34848), *Sxl* (BL-34393), *fl(2)d* (BL-55674), *vir* (BL-55694), *ime4* (BL-41590), *CG12076/YT521-B* (BL-34627) and *CG6422* (BL-55151). We induced mitotic clones by applying two 2 h heat-shocks at 37 °C at the end of L3. We induced flip-out clones expressing RNAi transgenes by applying a 30-minute heat-shock to 1- to 2-day old adult females. Ovaries were dissected 3–4 days after eclosion.

We fixed tissues in PBS containing 4% formaldehyde and incubated with the following primary antibodies: mouse anti-Hts (1:100, #1B1, DSHB), rabbit anti-GFP (1:1,000, Invitrogen, #11122), rabbit anti-Vasa (1:500, gift of Ruth Lehmann, NYU), mouse anti-Sxl (1:100, #M18, DSHB); mouse anti-HA (1:200, Santa Cruz, #Sc-7392), mouse anti-myc 9E10 (1:100, DSHB), and mouse anti-ß-galactosidase (1:100, DSHB). Secondary antibodies were Alexa-568 goat anti-mouse (1:1,000, Invitrogen #A-11031) and Alexa-488 anti-rabbit (1:1,000, Invitrogen, #A11034). Images were captured with a Leica SP5 confocal microscope.

### Analysis of m^6^A by quantitative mass spectrometry

We collected total RNA from *yw* (the genetic background used for CRISPR mutagenesis), *ime4* and *mettl14* homozygotes and hemizygous adult females, and subjected the samples to one round of polyA selection using the Oligotex kit (Qiagen). Before mass spectrometric analysis, all RNA samples were hydrolysed enzymatically to ribonucleosides. Briefly, the digestion was carried out in 0.1 mM Tris buffer (pH 8), containing 5 mM MgCl_2_, 0.0375 U per μl benzonase, 0.17 U per μl alkaline phosphatase, 1 U per ml phosphodiesterase, 1 μg per mL coformycin, 3 mM desferroxamine (antioxidant), 0.3 mM butylated hydroxytoluene (antioxidant), 0.05 μM [^15^N_5_]- 2′-deoxyadenosine (internal standard), and 5 μM 2′-deoxyinosine (internal standard) at 37 °C for 2 h. Enzymes were then removed using a YM-10 centrifugal spin column (Millipore).

Quantitative LC-MS analyses of m^6^A and m^6^A_m_ were achieved using an Agilent 1200 HPLC coupled to an Agilent 6430 triple quadrupole mass spectrometer in positive ion mode using dynamic multiple reaction monitoring. The ribonucleosides in the hydrolysed RNA samples were resolved on a Phenomenex C18 HPLC column (1.7 μm particle size, 100 Å pore size, 2.1 × 150 mm; 25 °C) at 330 μl min^−1^ using a solvent system consisting of 10 mM ammonium acetate in H_2_O (A) and acetonitrile (B). The elution profile was 0% B for 3 min, 0–7% B over 20 min, then to 7–40% B over 4 min, followed by a column washing at 80% B and column equilibration. The operating parameters for the mass spectrometer were as follows: gas temperature 350 °C; gas flow 10 L per min; nebulizer 50 psi; capillary voltage 3,500 V; fragmentor voltage 100 V; collision energy 15 V. The quantification of a ribonucleoside can be achieved using *m*/*z* of the parent ribonucleoside ion and *m*/*z* of the deglycosylated ion product. The nucleosides were quantified based on the transition of the parent ribonucleoside to the deglycosylated base ion: *m/z* 282.1→150.1 for m6A and *m/z* 268.1→136.1 for A. Absolute quantities of each ribonucleoside were determined using an external calibration curve prepared with synthetic standards.

### *Sxl* rt-PCR and RIP-rtPCR

We adapted a protocol from our recent study^34^. Plasmids of 5 μg were transfected into 6 × 10[6] S2 cells using Effectene (Qiagen) and incubated for 3 days. Cells were washed with PBS and lysed with IP lysis buffer (25 mM Tris–HCl, 150 mM NaCl, 1 mM EDTA, 1% NP40, 5% Glycerol) supplied with Complete, EDTA-free Protease Inhibitor and 40 U ml^−1^ SUPERase In RNase Inhibitor (Ambion) on ice for 30 min, followed by 2 × 10′ centrifugation at 20,000 *g* at 4 °C. 10% of the cleared cell lysate were kept as input and the rest was incubated with 15 μl anti-GFP nanobody agarose beads (Chromotek) for 2 h at 4 °C. The beads were washed three times using IP lysis buffer and then resuspended in 100 μl lysis buffer. To elute RNA, the beads were mixed with 900 μl of Trizol, vortexed for 1 min and incubated at RT for 5′ with rotation. RNA extracted and treated were Turbo DNase (Ambion) for 30 min before cDNA synthesis using SuperScript III (Life technology) with random hexamers. PCRs were done using Fusion High-Fidelity Polymerase (ThermoFisher Scientific). *Sxl* and *Rpl32* primer sets are listed in Supplementary Data 7.

### Data availability

All the raw sequence data for miCLIP and RNA-seq datasets are available at the NCBI Gene Expression Omnibus under GSE97004.

## Additional information

**How to cite this article:** Kan, L. *et al*. The m^6^A pathway facilitates sex determination in *Drosophila*. *Nat. Commun.* **8,** 15737 doi: 10.1038/ncomms15737 (2017).

**Publisher’s note:** Springer Nature remains neutral with regard to jurisdictional claims in published maps and institutional affiliations.

## Supplementary Material

Supplementary Information Supplementary Figures

Supplementary Data 1 miCLIPand mRNA-seqlibrary stats.

Supplementary Data 2 RAC/DRACH enrichment stats in miCLIP libraries.

Supplementary Data 3 Summary of genomic locations of CIMS/m6A loci.

Supplementary Data 4 Catalog of genic CIMS/m6A sites inferred from miCLIP libraries.

Supplementary Data 5 GO analysis of genes with miCLIP enrichment and CIMS sites.

Supplementary Data 6 Catalog of intronic CIMS/m6A sites inferred from miCLIP libraries.

Supplementary Data 7 Primers used in this study.
