# Supplementary material for: The m6A pathway facilitates sex determination in Drosophila
Source: Nat Commun. 2017 Jul 4;8:15737. doi: 10.1038/ncomms15737 (PMC5500889; doi:10.1038/ncomms15737)
Supplement: Supplementary Information — Supplementary Figures [file ncomms15737-s1.pdf]

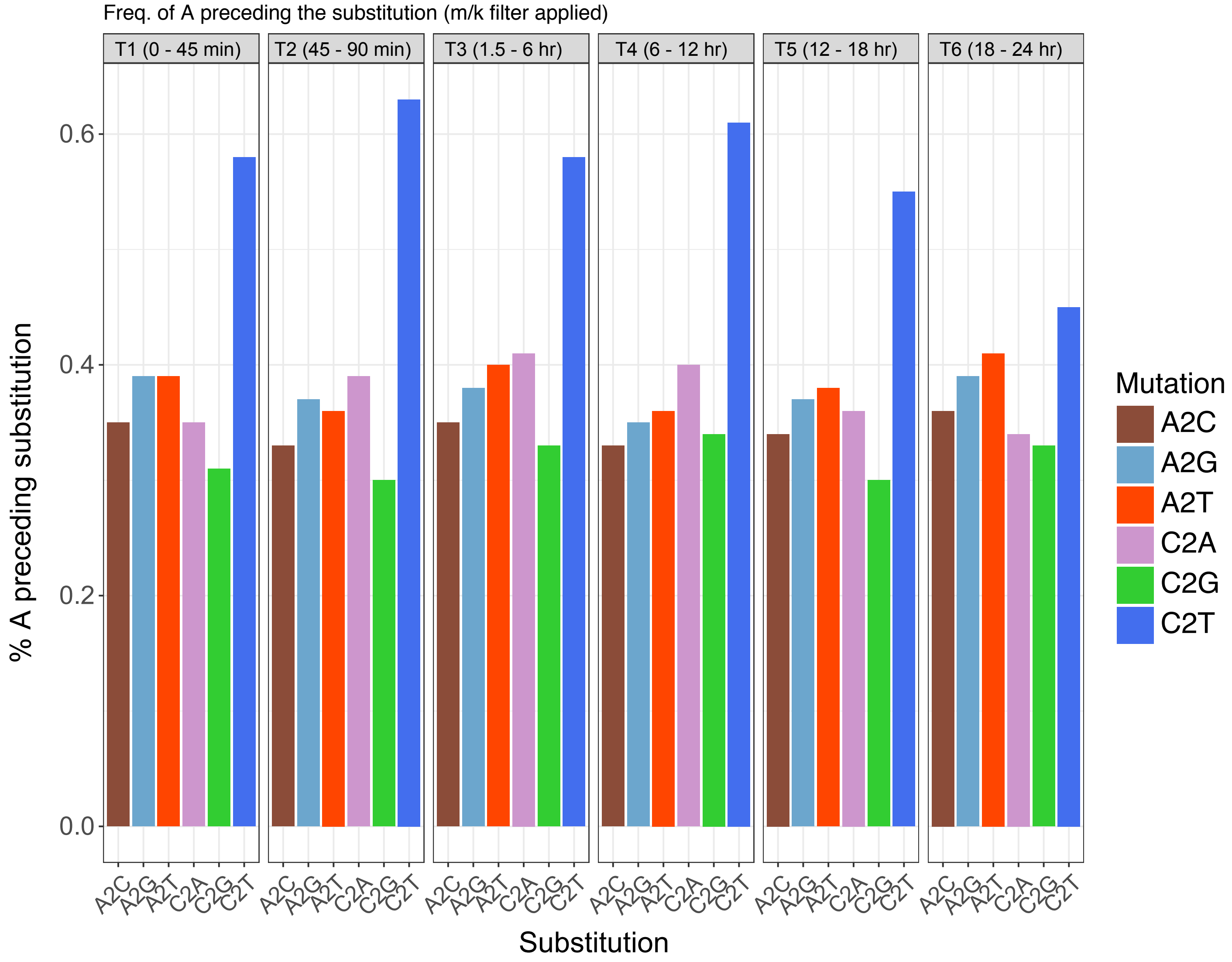

Supplementary Figure 1. Selective enrichment of A upstream of C-to-T transitions in miCLIP data.
The frequency of adenosine preceding C-to-T transitions annotated through the CIMS pipeline is substantially higher than other types of nucleotide substitutions. Note that for the T6 library, the "A" enrichment is not as high as in other libraries, indicating CIMS confidence is less certain.

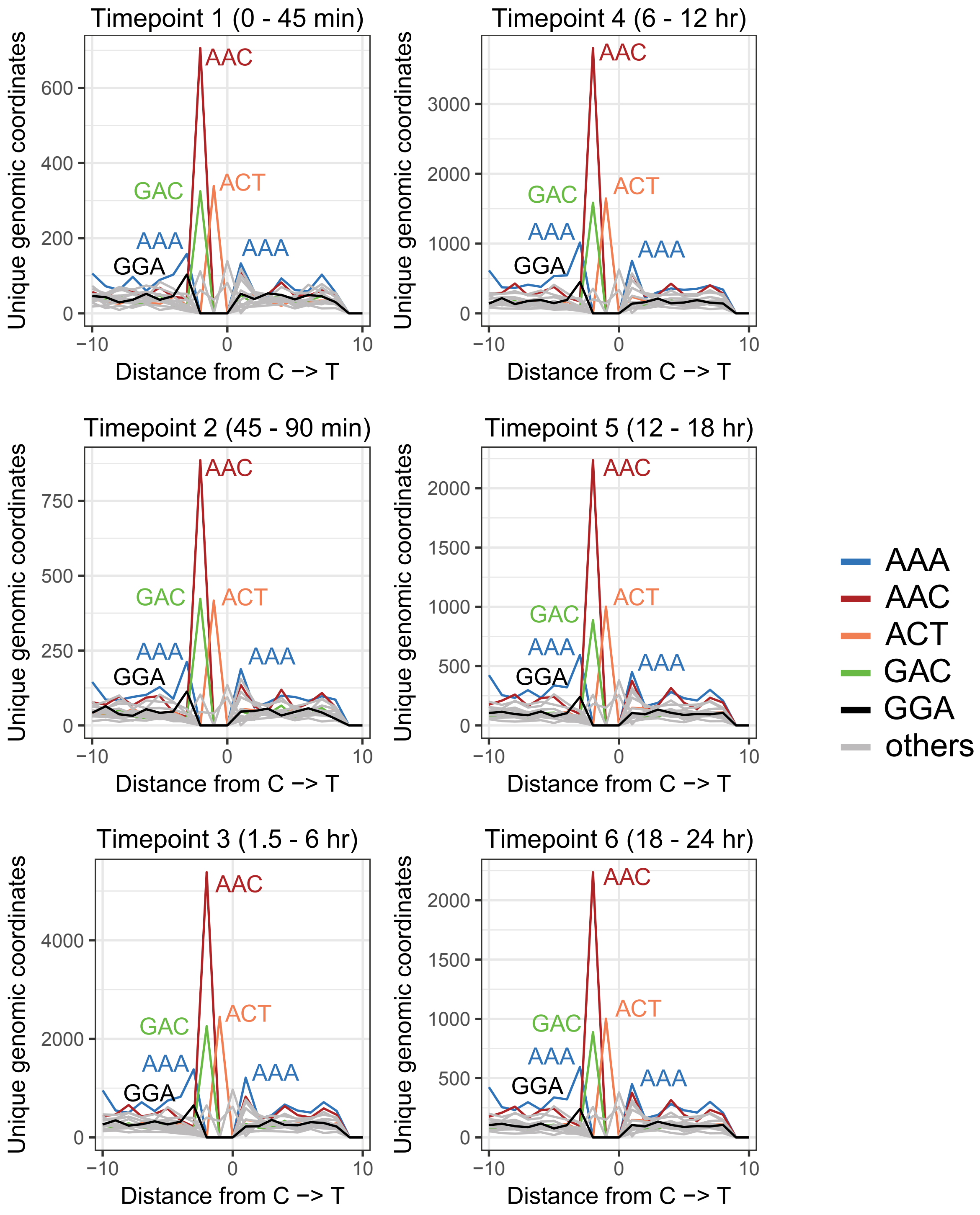

Supplementary Figure 2. Positional enrichment of submotifs in the vicinity of CIMS calls.
Shown are motif enrichment plots centered on CIMS calls in each library. The peak is shown at the first nucleotide of the motif. GAC, AAC, and ACT, which form submotifs on the canonical DRACH motif of m6A sites in other species. Note that the fly m6A sites show preference for being in local A-rich regions (AAA submotifs, which are not contingent on location at a CIMS). However, GGA submotif, which is also not contingent on location at a CIMS, is also enriched precisely at the three nucleotides upstream of CIMS.

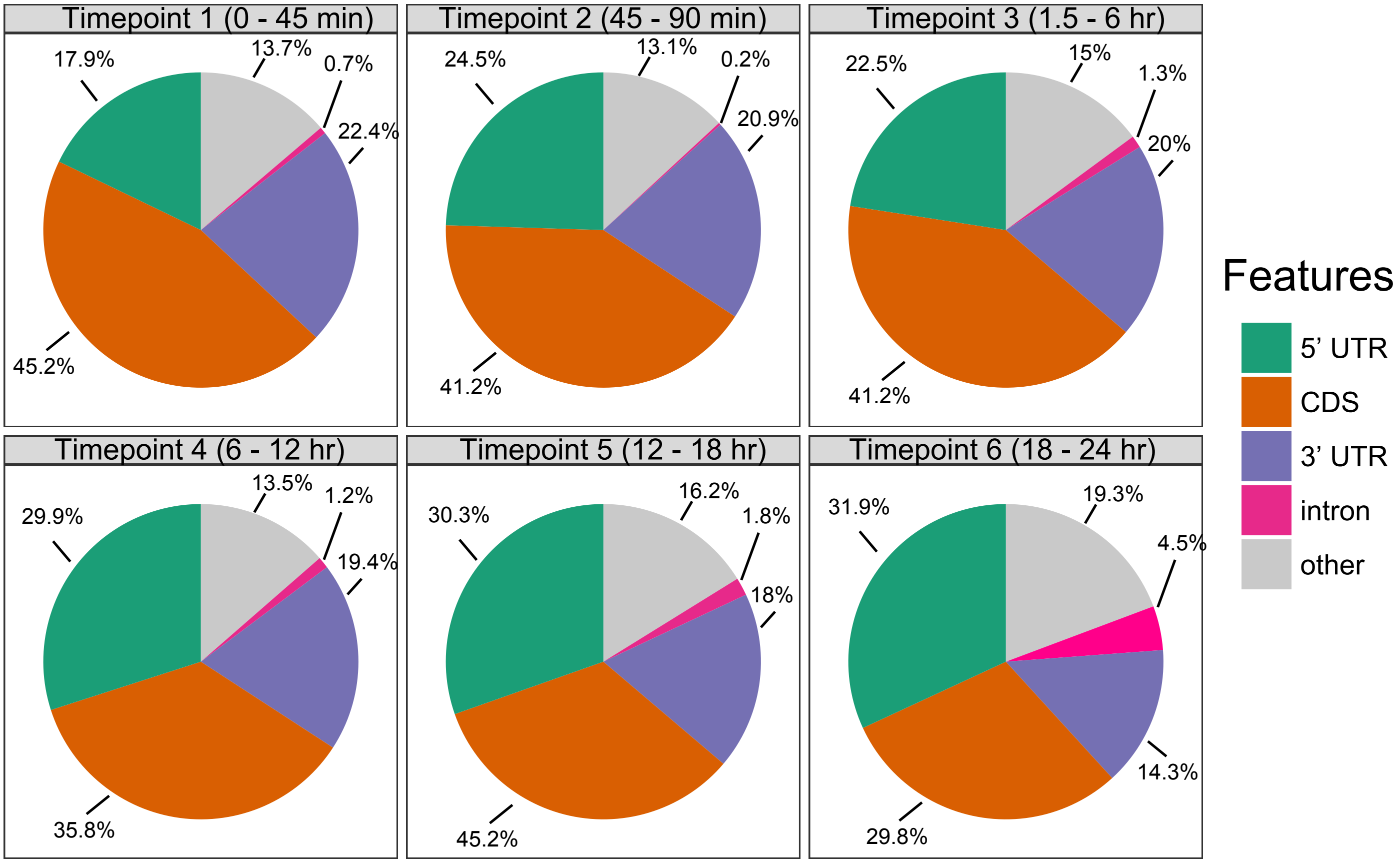

Supplementary Figure 3. Genomic locations of CIMS calls across the miCLIP datasets.

The overall distribution of CIMS calls relative to genomic annotations is similar across embryogenesis, except that the fraction of intronic hits increases with the onset of zygotic transcription (in timepoint 3).

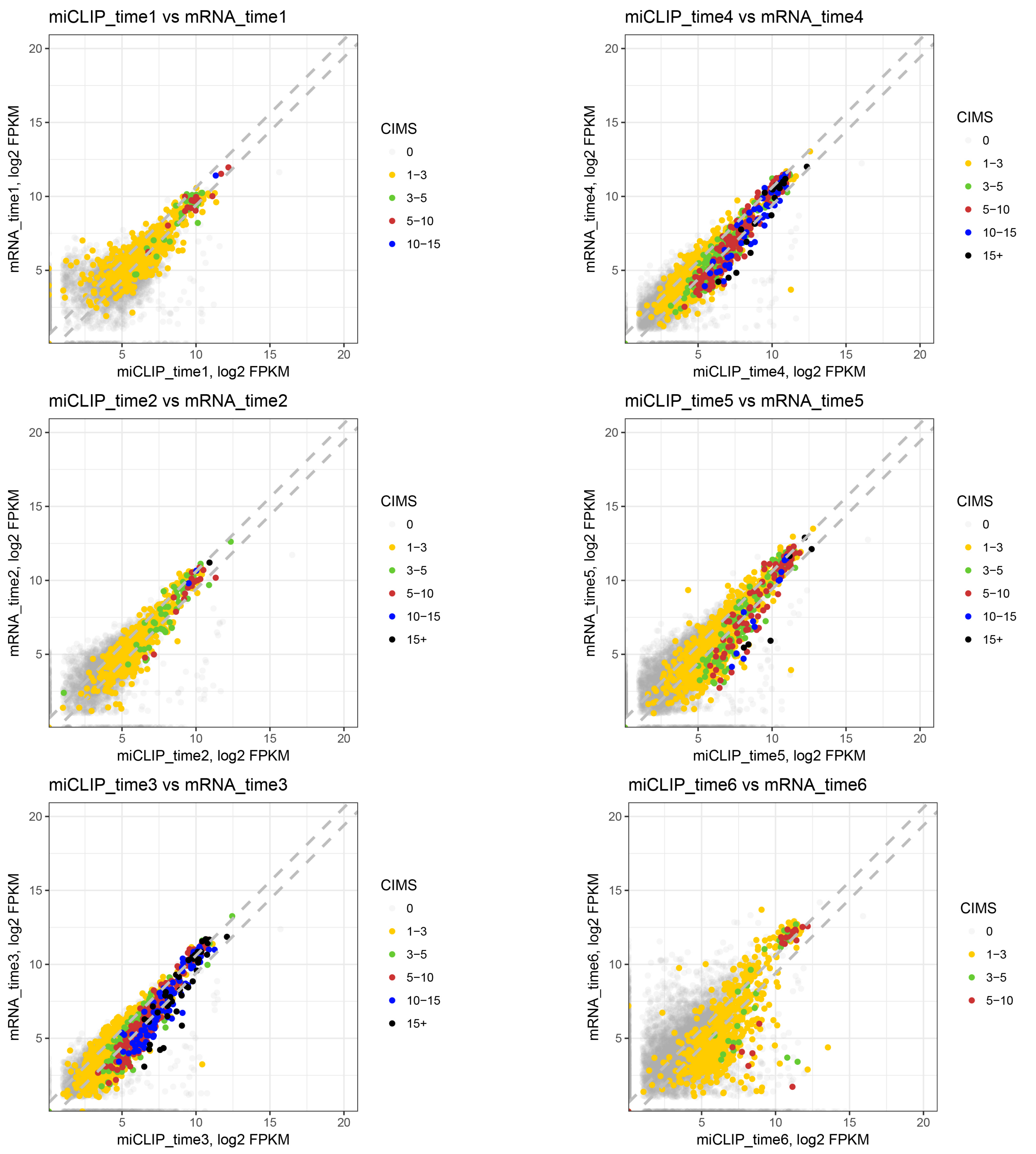

Supplementary Figure 4. miCLIP vs mRNA-seq correlations.

Correlation of miCLIP and RNA-seq expression across the 6 developmental timepoints across embryogenesis. We grouped comparable modENCODE mRNA-seq datasets for this analysis. The dotted lines indicate 1.5-fold difference, and the number of CIMS sites per gene is color-coded. In general, there is a good correlation between miCLIP and RNA-seq data, but a skew towards miCLIP enrichment can be seen for a subset of loci in each library.

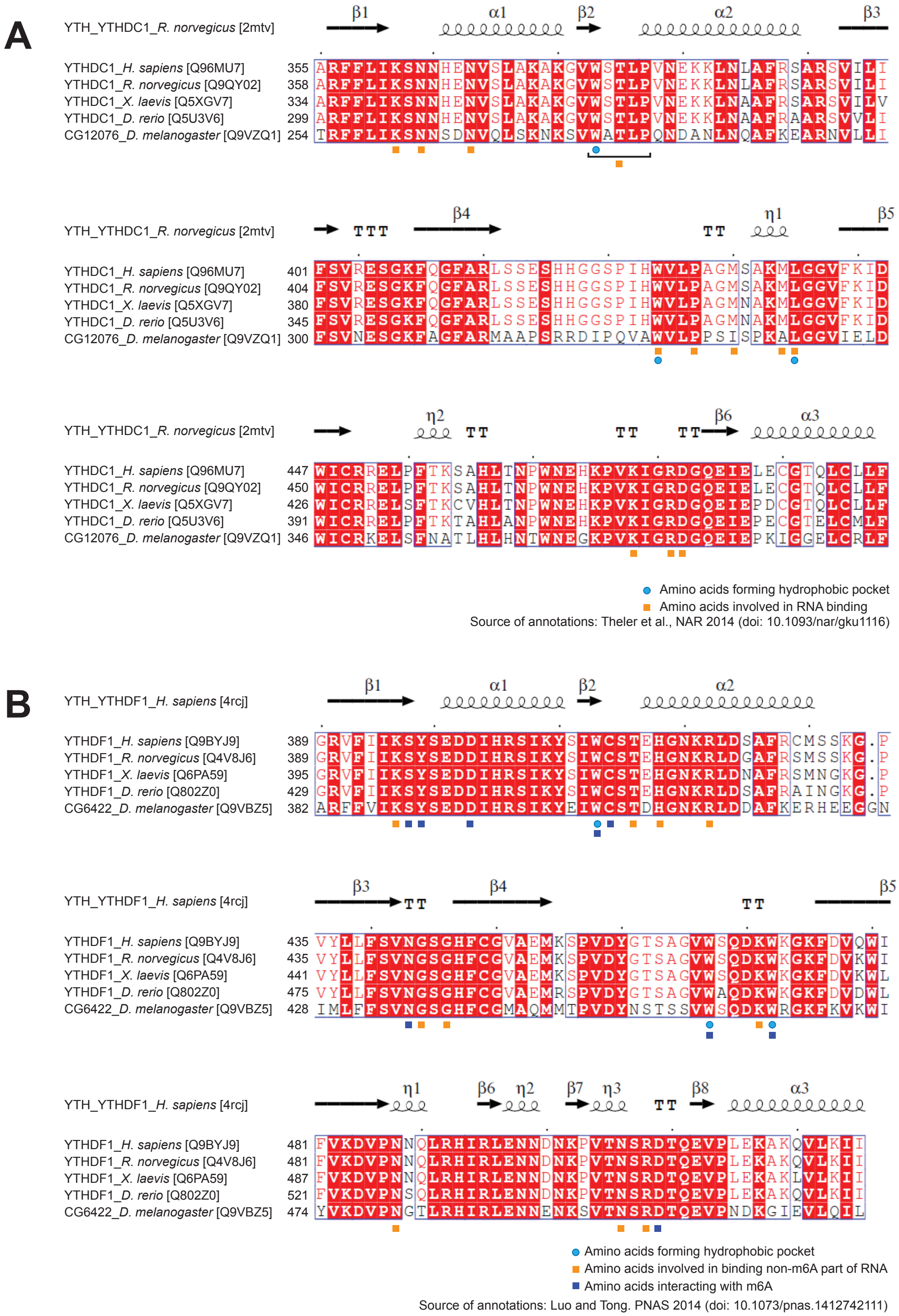

Supplementary Figure 5. Drosophila YTH domains bear conserved features for m6A binding.

YTH domains from CG12076/YT521-B (A) and CG6422 (B) have a significant homology with the domains from other YTH proteins that are known to bind m6A. Shown here are amino acid alignments of the two YTH domains with the YTH domains from other organisms. The secondary structure elements from rat YTHDC1 (PDB accession number: 2mtv) and human YTHDF1 (PDB accession number: 4rcj) are also shown at the top of each alignment in panels A and B, respectively. Amino acids interacting with m6A and the RNA backbone are indicated below the alignments with colored circles and squares. Residues forming the hydrophobic pocket are indicated by blue circles, and the residues contacting the RNA are shown in orange or blue squares.

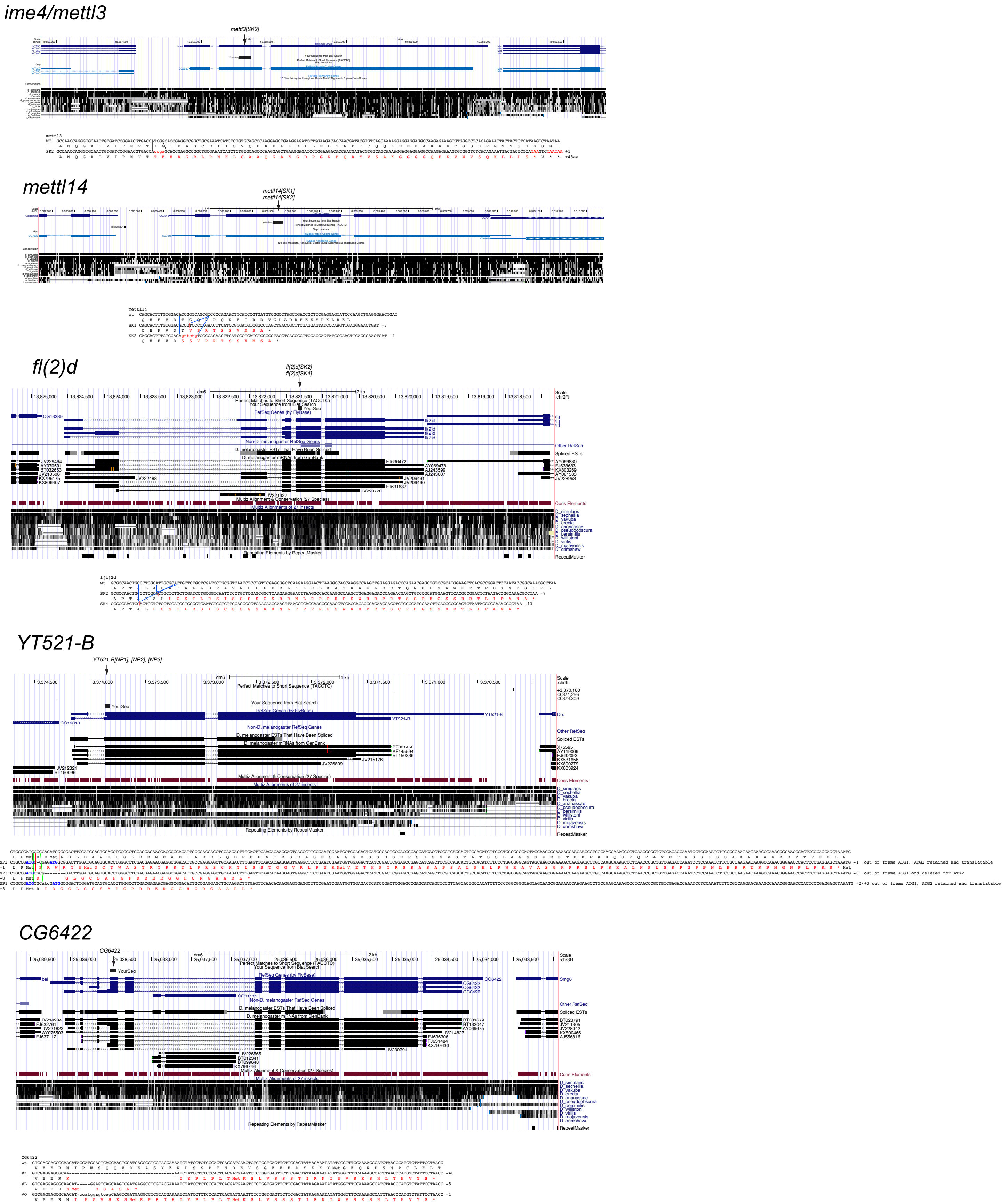

Supplementary Figure 6. CRISPR-induced mutations in m6A pathway factors.

Shown are genomic details of the five m6A factors subjected to CRISPR/Cas9-mediated mutagenesis. The locations of frame-shift indels are indicated above each locus, and the genomic alterations and predicted mutant proteins are shown below each locus.

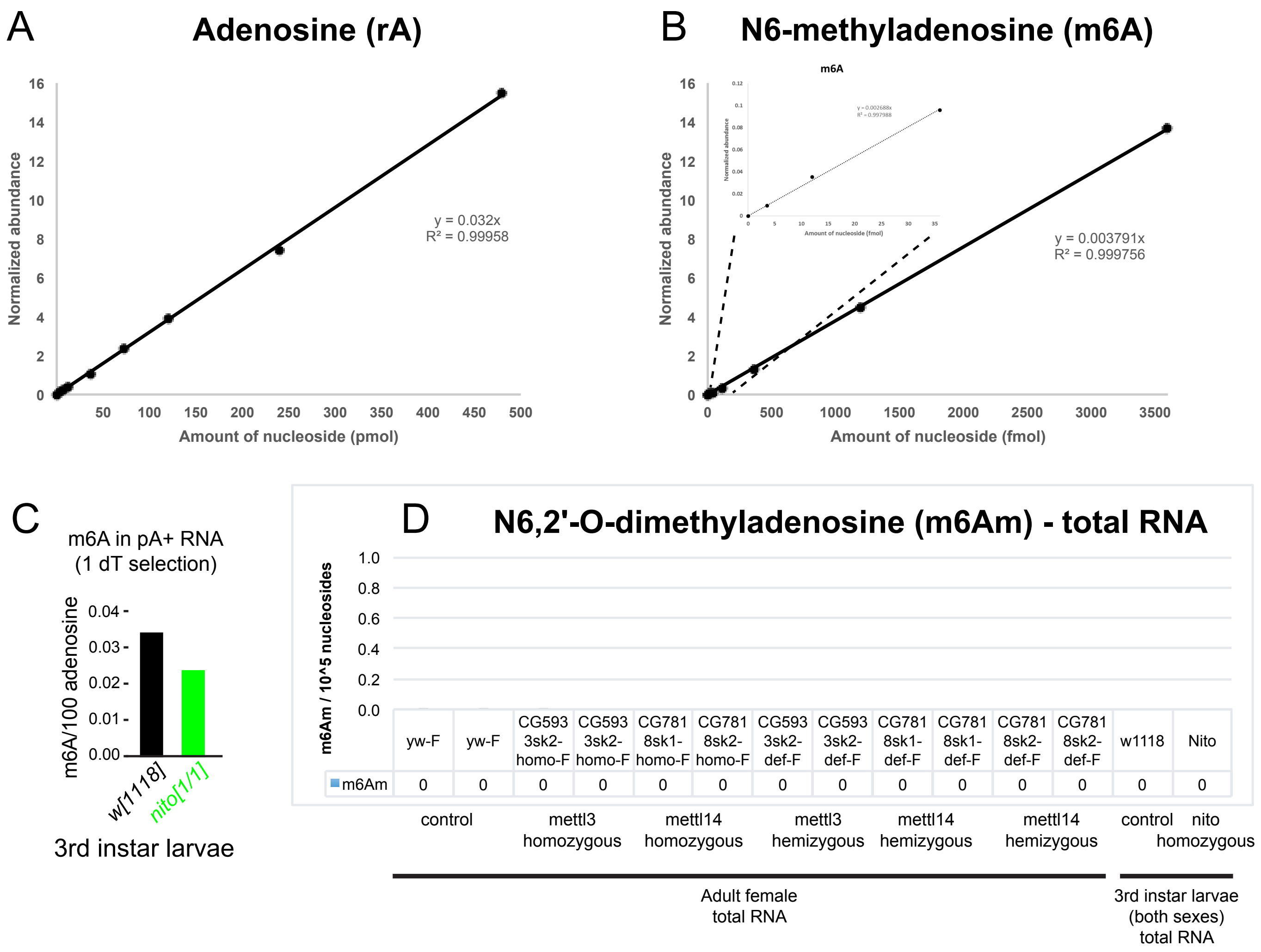

Supplementary Figure 7. Calibration curves for m6A quantification and m6Am analysis.

Shown are standard calibration curves for detection of adenosine (A) and N6-methyladenosine (B) used for absolute quantifications. Inset on (B) shows the lower quantification range. (C) Modest reduction in m6A in nito homozygous mutant larvae compared to control w[1118]. The modest reduction might be due to maternal deposits and/or incomplete rRNA depletion by single dT selection. (D) Failure to detect m6Am in total RNA of control and m6A pathway mutants.

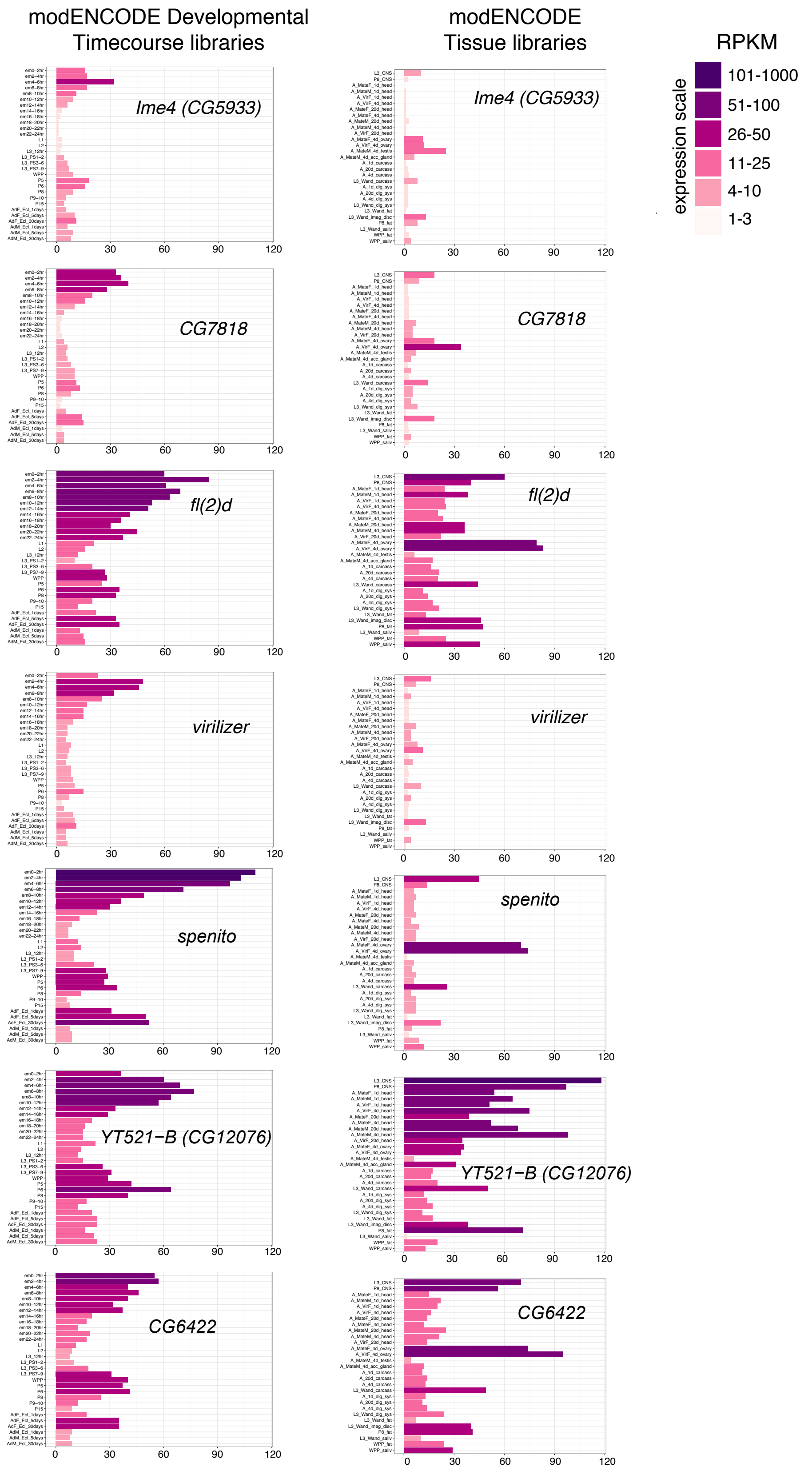

Supplementary Figure 8. Expression of m6A-related factors across modENCODE data.

Shown are the RPKM measurements of the indicated factors across diverse developmental time-course datasets (left column) or dissected tissue datasets (right column) produced by the modENCODE project. Note that all factors exhibit maximal or preferential expression in neural libraries (e.g., larval or pupal CNS, or adult heads), and that gonads (ovaries or testes) represent another location of elevated expression of m6A-related factors.

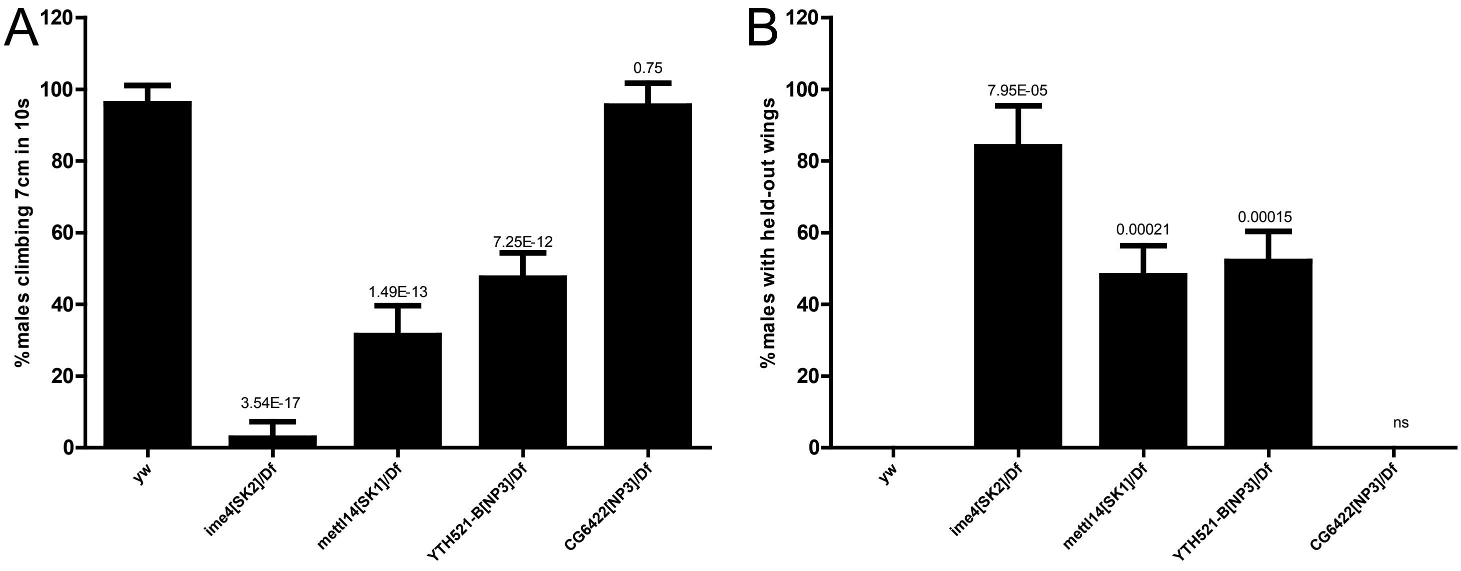

Supplementary Figure 9. Defective negative geotaxis and held-out wings in m6A-pathway mutant males. (A) Negative geotaxis assay. 10 flies were placed in an empty vial and tapped to the bottom, and their ability to climb was quantified. Five independent cohorts of flies per genotype were assays, and the assay was done in triplicate for each group of flies. Nearly all control (yw) flies cross the 7cm mark within 10 seconds; indeed, nearly all of these reached this mark in <5 seconds. Most ime4 hemizygotes stayed at the bottom of the vial, and a minority slowly climb to the designated height. mettl14 and YT521-B hemizygous mutants also display strongly reduced negative geotaxis, whereas CG6422 hemizygotes were normal. (B) Quantification of held-out wings in the indicated genotypes. This wing posture defect is roughly correlated with the presence of locomotor defects quantified in other assays.

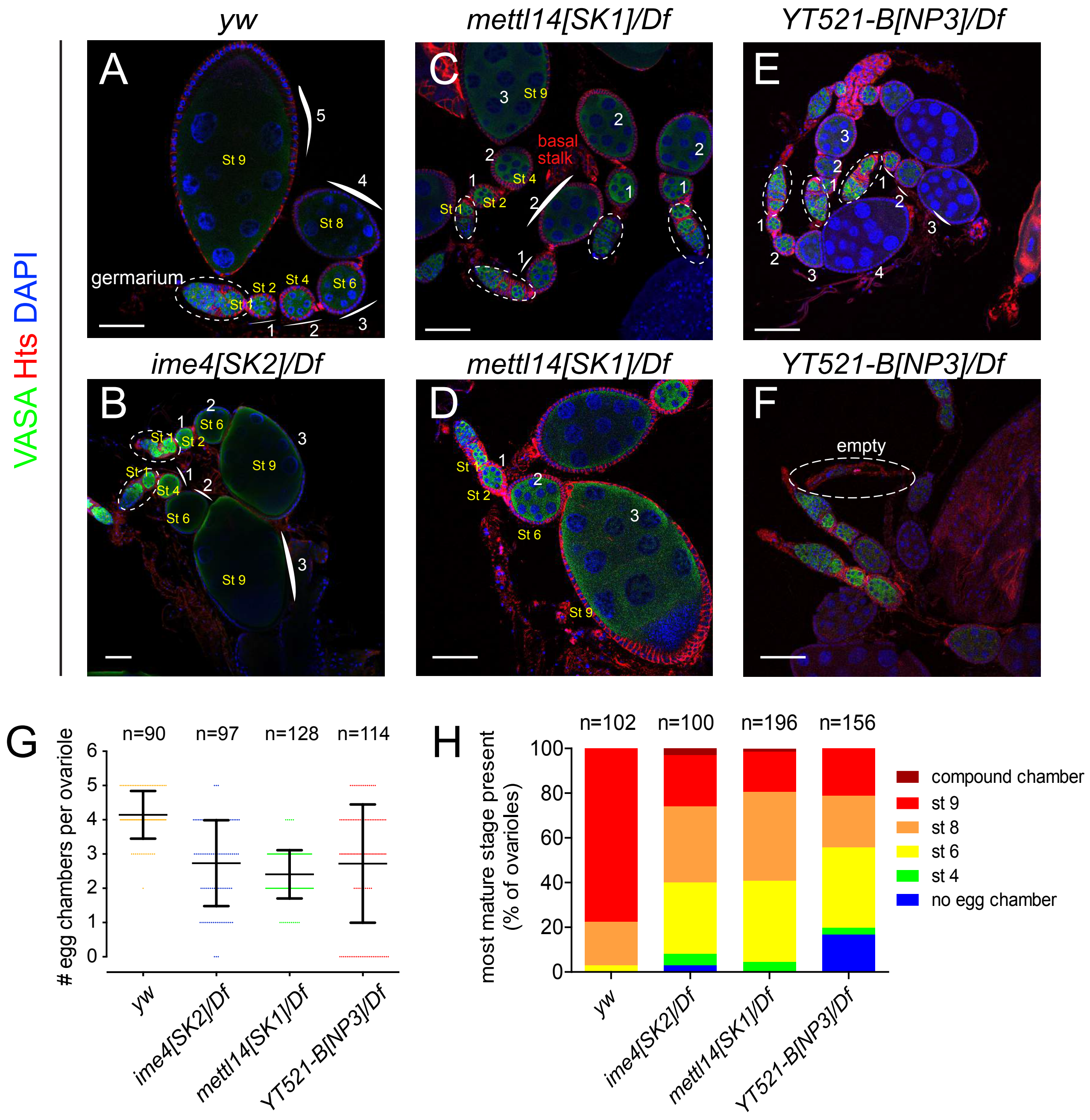

Supplementary Figure 10. Oogenesis phenotypes of m6A pathway mutants

(A–F) Ovaries were stained with anti-Hts (red) and anti-Vasa (green) antibodies. Germarium structures are marked with dotted outlines, and the numbers of egg chambers in selected individual ovarioles are labeled with white numbers. The stages of selected egg chambers are labeled in yellow. (A) Control yw genotype illustrates the normal progression of an ovariole with a germarium followed by a string of egg chambers of various stages, in this case five egg chambers going up to stage 9. (B) ime4 hemizygous ovarioles showing abbreviated sets of egg chambers. (C-D) Examples of mettl14 hemizygous ovarioles, illustrating either abbreviated strings of egg chambers (C) or an ovariole that contains a relatively mature stage 9 egg chamber but misses some earlier stages (D). (E-F) Examples of YT521-B hemizygous ovarioles, which either show mild loss of egg chambers (E) or a rarer class of empty ovariole lacking egg chambers (F). scale bars, 50 μm. (G) Quantification of egg-chamber number shows that different m6A pathway mutant ovaries exhibit fewer than in yw control. (H) Distribution of egg chamber stages shows a skew towards early stages in m6A pathway mutants relative to yw control.

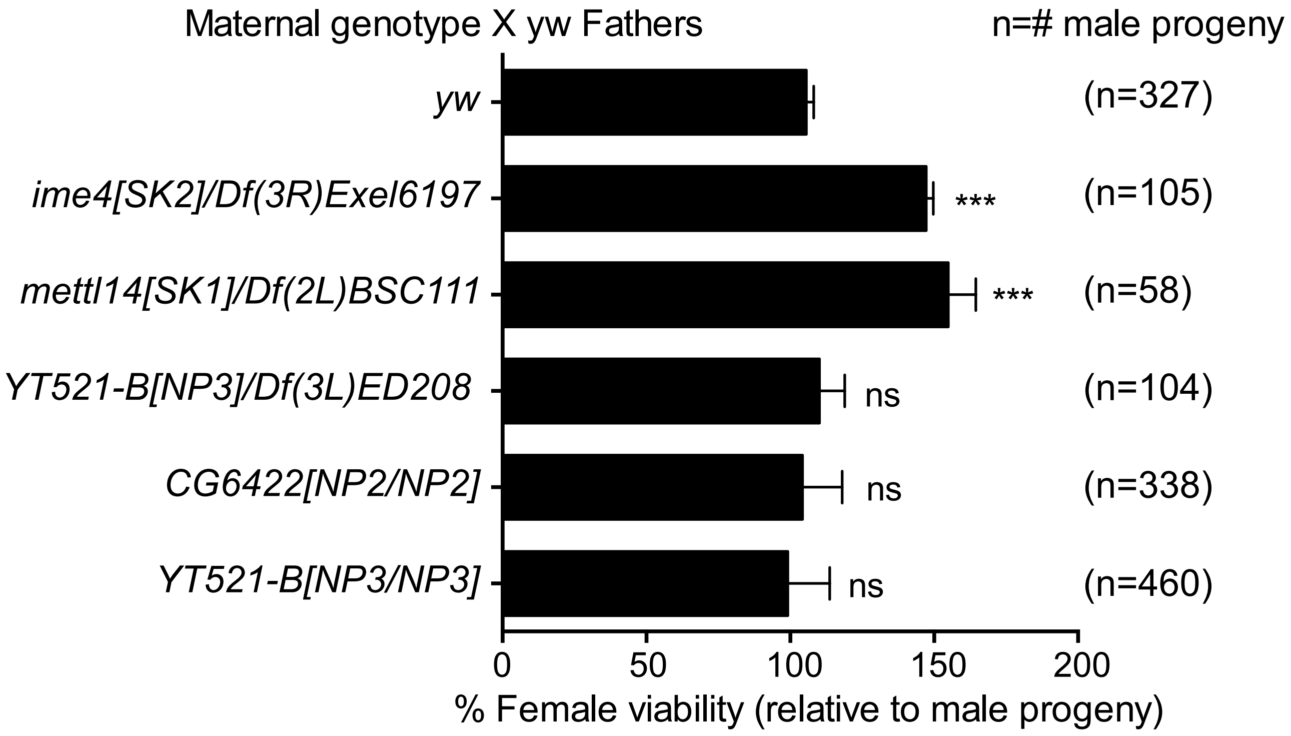

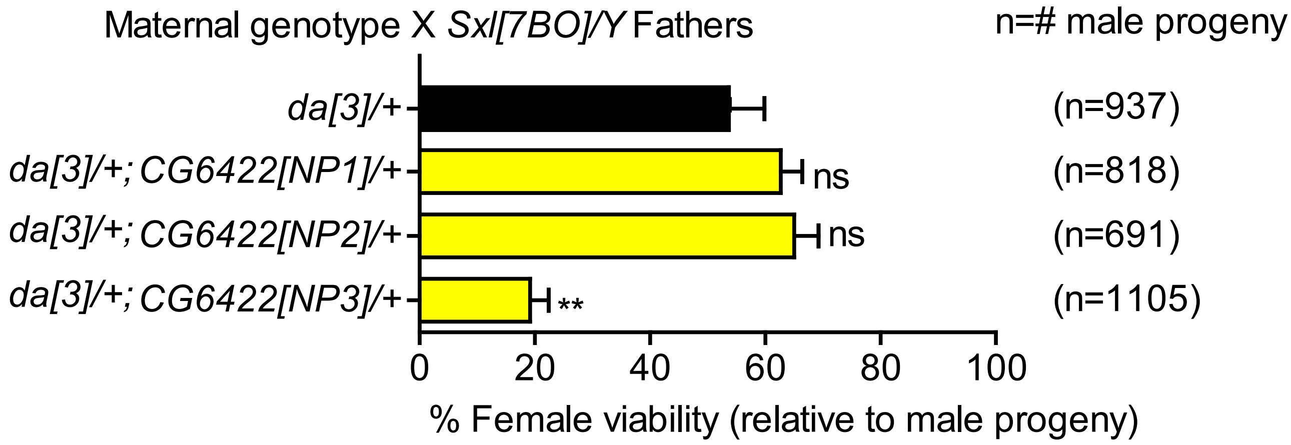

Supplementary Figure 11. Additional analysis of female survival in m6A pathway mutants.

(Top) This graph summarizes the female survival as a percentage of male siblings in various m6A pathway mutants. Female lethality is not observed, in contrast to genetic sensitization experiments involving *Sxl* heterozygosity.

(Bottom). Genetic interaction tests of *Sxl*, *da* and *CG6422*. Maternal heterozygosity of *da* combined with paternal *Sxl* heterozygosity results in ~50% female lethality. This was not modified by two of the *CG6422* alleles, but inclusion of *CG6422[NP3]* dominantly enhanced female lethality.

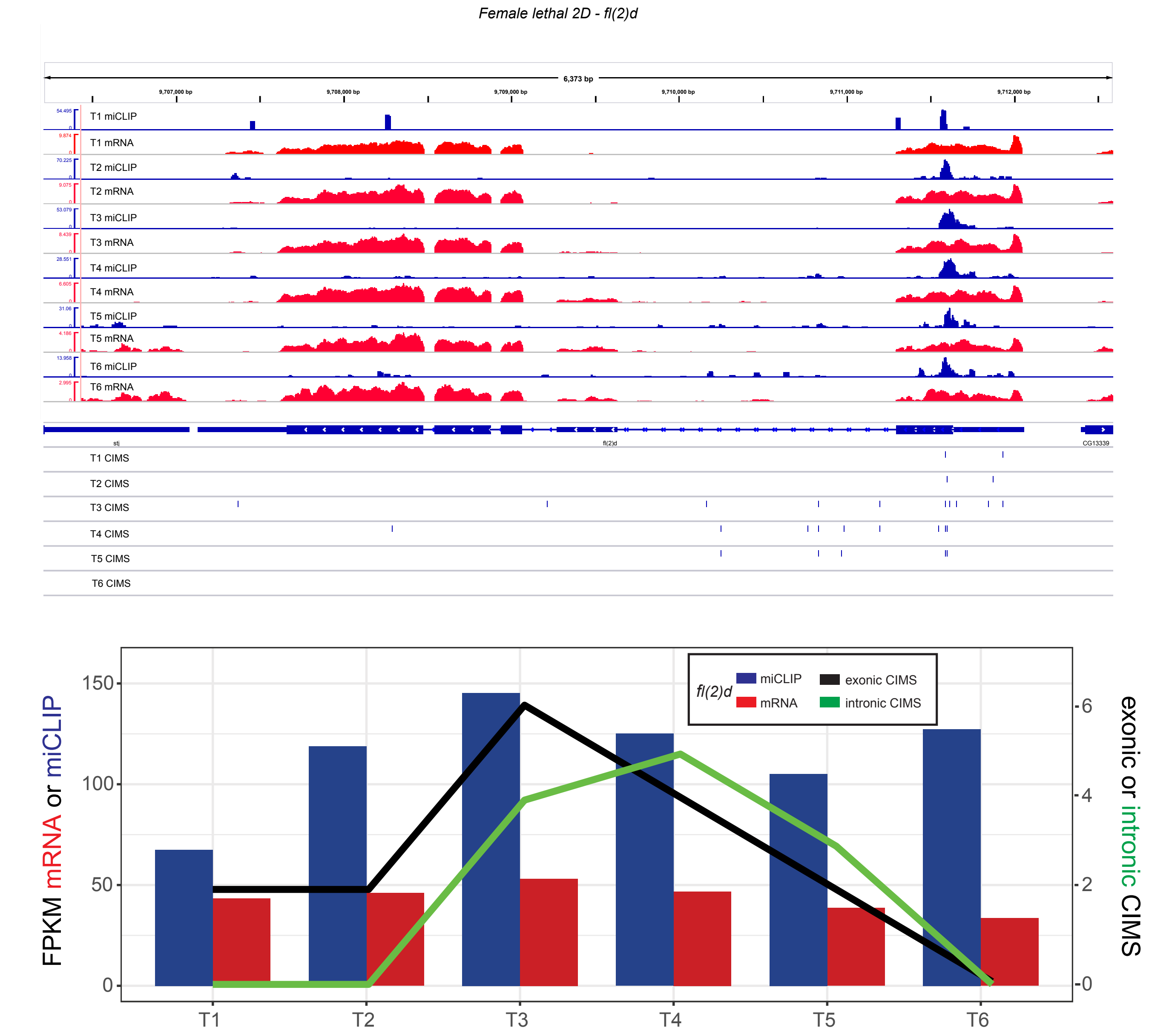

Supplementary Figure 12. Summary of mRNA-seq, miCLIP and CIMS calls at fl(2)d.

Along with Sxl (Figure 7C-D), fl(2)d is one of the top loci in the genome in terms of intronic CIMS calls (see also Supplementary Table S6). (Top) Summary of miCLIP, mRNA-seq and CIMS tracks at fl(2)d. A prominent set of CIMS calls is observed in its first intron, in addition to a 5' UTR peak. (Bottom) Analysis of RNA-seq and miCLIP expression (left Y-axis), and exonic/intron CIMS sites (right Y-axis) across embryogenesis. These summaries emphasize that miCLIP and CIMS calls at f(l)2d are found specifically in datasets following activation of zygotic transcription.

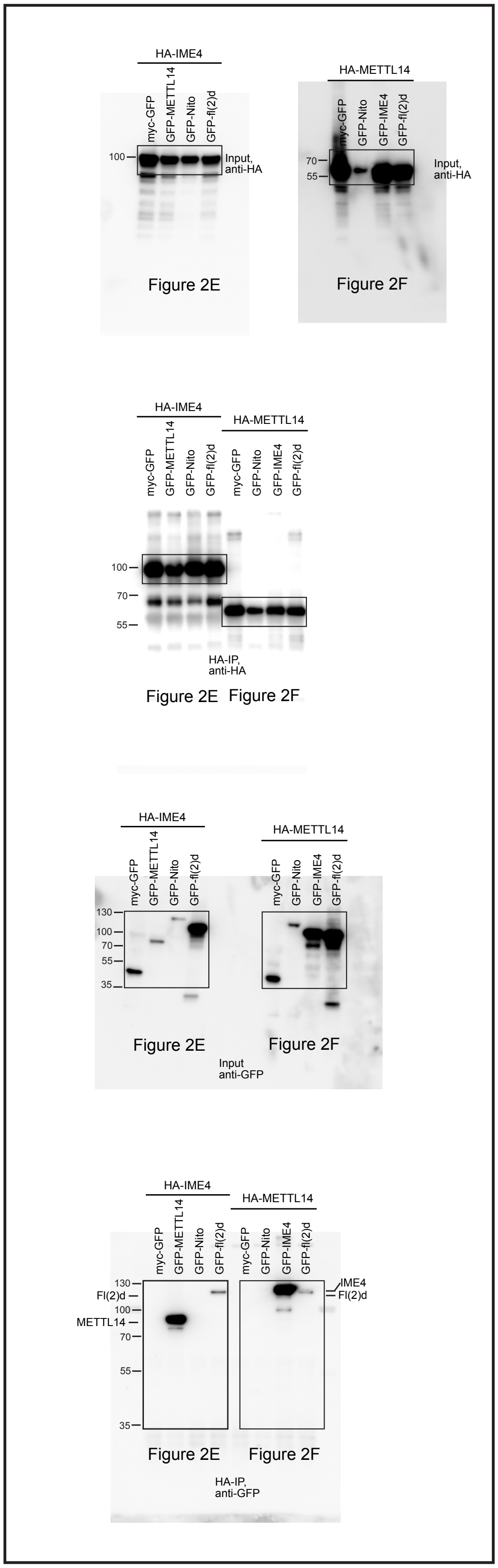

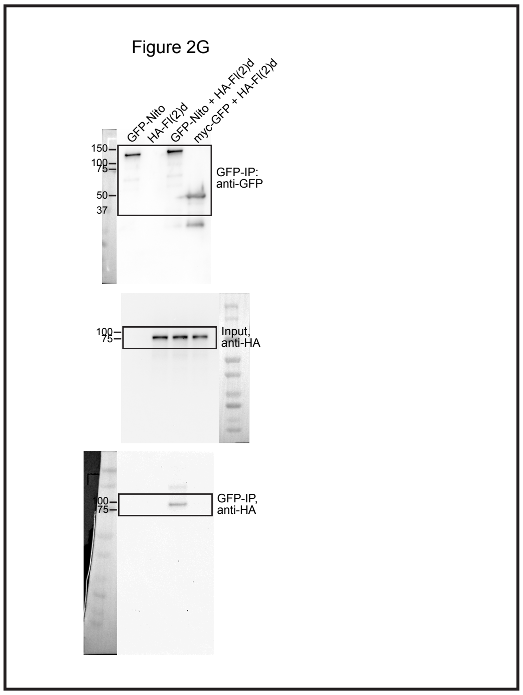

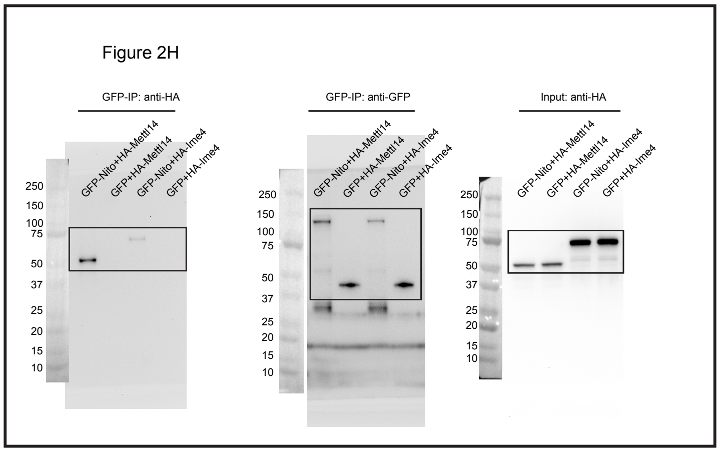

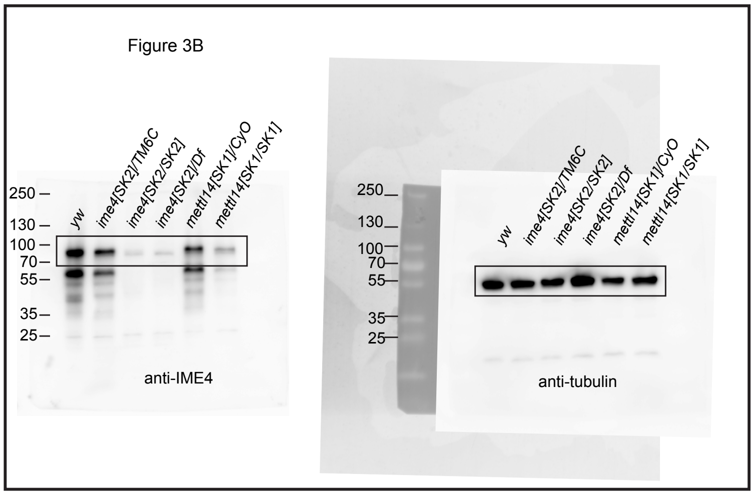

Supplementary Figure 13. Uncropped Western blots for data presented in the main figures.
